# Trap**α** deficiency impairs the early events of insulin biosynthesis and glucose homeostasis

**DOI:** 10.1172/JCI179845

**Published:** 2025-05-20

**Authors:** Xin Li, Jingxin Hu, Yumeng Huang, Hai Zhang, Ning Xu, Yang Liu, Xuan Liu, Yuanyuan Ye, Xinxin Zhang, Xiaoxi Xu, Yuxin Fan, Ziyue Zhang, Weiping J. Zhang, Shusen Wang, Wenli Feng, Peter Arvan, Ming Liu

**Affiliations:** 1 Department of Endocrinology and Metabolism, Tianjin Medical University General Hospital, Tianjin, China.; 2 National Key Laboratory of Immunity and Inflammation, Department of Pathophysiology, Naval Medical University, Shanghai, China.; 3 Human Islet Resource Center, Tianjin First Central Hospital, Tianjin, China.; 4 Division of Metabolism, Endocrinology & Diabetes, University of Michigan Medical Center, Ann Arbor, Michigan, USA.

**Keywords:** Endocrinology, Metabolism, Beta cells, Insulin, Mouse models

## Abstract

Defects in the early events of insulin biosynthesis, including inefficient preproinsulin (PPI) translocation across the membrane of the ER and proinsulin (PI) misfolding in the ER, can cause diabetes. Cellular machineries involved in these events remain poorly defined. Genes encoding translocon-associated protein α (TRAPα) show linkage to glycemic control in humans, though their pathophysiological role remains unknown. Here, we found that β cell–specific TRAPα-KO mice fed a chow diet or a high-fat diet (HFD) had decreased levels of circulating insulin, with age- and diet-related glucose intolerance. Multiple independent approaches revealed that TRAPα-KO not only causes inefficient PPI translocation but also leads to PI misfolding and ER stress, selectively limiting PI ER export and β cell compensatory potential. Importantly, decreased TRAPα expression was evident in islets of wild-type mice fed the HFD and in patients with type 2 diabetes (T2D). Furthermore, TRAPα expression was positively correlated with insulin content in human islet β cells, and decreased TRAPα was associated with PI maturation defects in T2D islets. Together, these data demonstrate that TRAPα deficiency in pancreatic β cells impairs PPI translocation, PI folding, insulin production, and glucose homeostasis, contributing to its genetic linkage to T2D.

## Abstract 2

<strong>Trapα deficiency impairs the early events of insulin biosynthesis and glucose homeostasis</strong>

## Introduction

Insulin (INS) is a master hormone regulating energy metabolism. In pancreatic cells, INS derives from its precursors, preproinsulin (PPI) and proinsulin (PI). Newly synthesized PPI, led by its N-terminal signal peptide, undergoes co- and posttranslational translocation across the membrane of the ER via the Sec61 translocon (1, 2). Upon entering the lumen of the ER, the PPI signal peptide is excised by signal peptidase, forming PI that rapidly undergoes oxidative folding to form 3 intramolecular disulfide bonds that are critical for achieving PI native structure, leading to its anterograde transport from the ER to the Golgi complex (3–6). Over the past 2 decades, increasing genetic and biological evidence has indicated that multiple factors can affect these early events of INS biosynthesis, and defects in these events can lead to cell failure and diabetes both in humans and animal models (7–11).

Insights into these processes have emerged from studies of monogenic diabetes caused by INS gene mutations. To date, more than 90 INS gene mutations have been identified to cause diabetes in humans (7). Among those mutations that have been experimentally tested, more than 90% cause diabetes by impairing either PPI translocation into the ER (2, 12), or signal peptide cleavage (3, 13), or PI folding in the ER (4, 8, 14, 15). These impairments not only directly decrease PI/INS production, they may also lead to abnormal intracellular accumulation of INS precursors (including PPI and/or PI) that may affect coexpressed WT bystander PPI and/or PI and may induce ER stress and cell toxicity, all of which can contribute to the development and progression of diabetes (1, 10, 16). Importantly, WT PPI and PI are predisposed to inefficient translocation and disulfide mispairing during oxidative folding (2, 12, 17). Moreover, defective PI folding and maturation have been found in *db/db* mice as well as in patients with type 2 diabetes (T2D) (18–20), suggesting that defects in early events of INS biosynthesis may contribute to the development and progression of mutant *INS* gene–induced diabetes and T2D.

In addition to PPI and PI molecules themselves, previous studies showed that dysfunction of the ER folding environment due to deficiency of key ER machineries can impede oxidative folding of PI (21–23). Recently, we showed that the translocon-associated protein (TRAP; also called signal sequence receptor [SSR]) complex, composed of 4 subunits (named TRAP/SSR1, TRAP/SSR2, TRAP/SSR3, and TRAP/SSR4), is critical for INS biosynthesis in pancreatic cell lines and mouse islets (24–27). Importantly, GWAS from multiple populations show that polymorphisms in the locus of TRAPα/SSR1 genes are associated with glycemic control defects, including fasting glucose, gestational diabetes, and T2D (28–30). However, to date, no evidence directly links those risk variants with the expression or function of TRAPα/SSR1 in β cells. Therefore, further studies examining the connections between the variants and expression of TRAPα/SSR1 in β cells are needed to determine whether TRAPα is a (or the only) causal gene at this locus associated with T2D.

Furthermore, although TRAPα/SSR1 is important for INS biosynthesis in β cell lines (26), no direct evidence shows the pathophysiological role of TRAPα in vivo. Here, we examined the role of TRAP in INS biosynthesis and glycemic control in whole animals. For this purpose, we established a β cell–specific TRAP-KO mouse line and found that TRAP deficiency causes INS deficiency with age- and diet-related glucose intolerance. Functional analysis revealed that TRAPα-KO not only causes PPI translocation inefficiency but also impairs PI oxidative folding, which limits PI ER export and INS production and induces β cell ER stress and apoptosis. Interestingly, we found that TRAPα expression is positively correlated with INS content in human islet β cells, and decreased TRAPα is associated with PI maturation defects in T2D islets, as well as in mice fed an HFD. These data demonstrate that TRAPα is critical for PPI translocation, PI folding, and INS production, contributing to its role in blood glucose homeostasis.

## Results

### TRAPα is highly expressed in human and mouse pancreatic cells; TRAPα-KO decreases circulating INS, resulting in age-related glucose intolerance.

**Figure 1:**
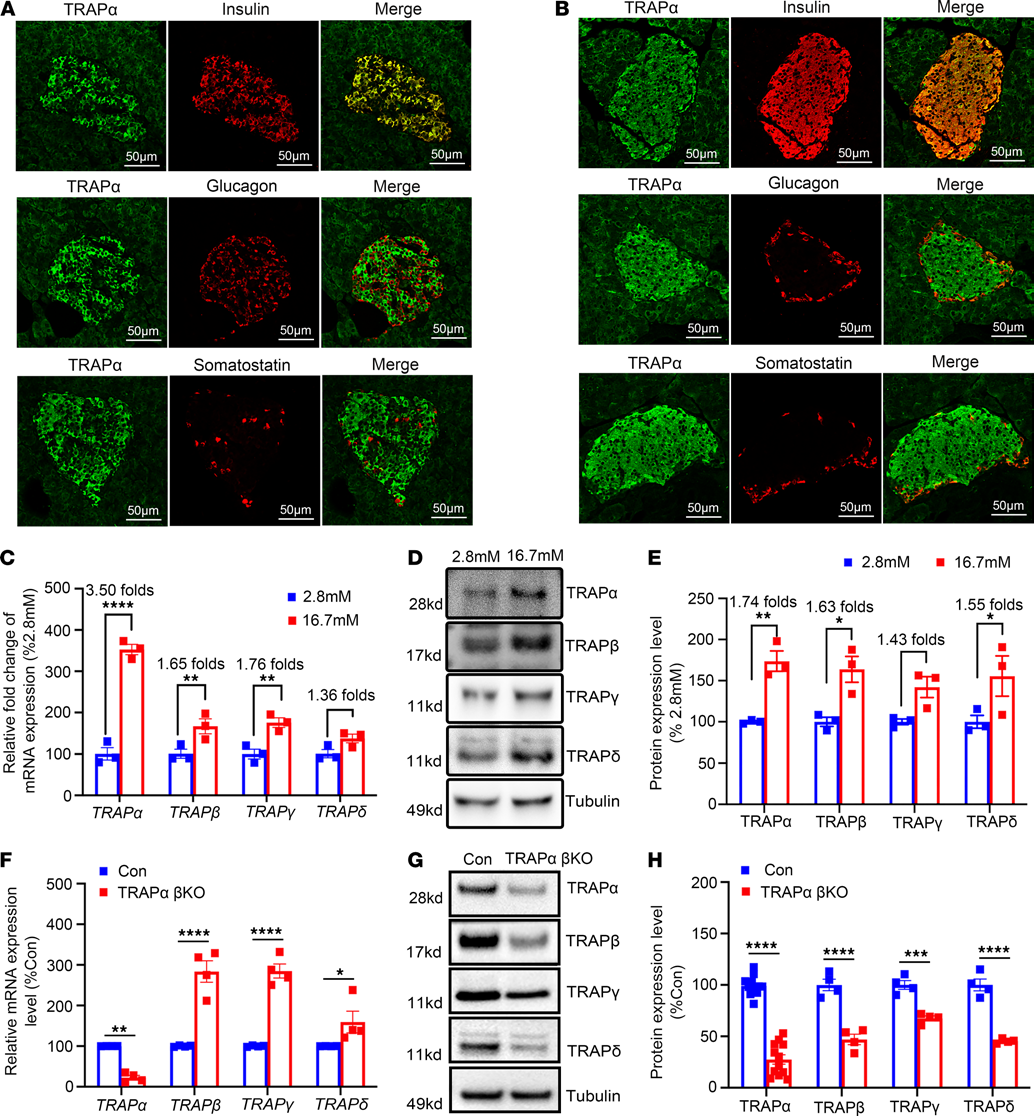
TRAPα is highly expressed in human and mouse pancreatic cells, and its expression is regulated by glucose. (**A** and **B**) The expression of TRAPα in human (**A**) and mouse (**B**) pancreases was detected via immunostaining with anti-TRAPα (green) and anti-INS (red), anti-GCG (red), or anti-somatostatin (red). (**C**) The islets isolated from 8- to 12-week-old WT mice were incubated in medium with either 2.8 mM or 16.7 mM glucose for 4 hours. The expression of mRNA of TRAPα, TRAPβ, TRAPγ, and TRAPδ was examined by real-time quantitative PCR. The mRNA levels in islets incubated with 2.8 mM glucose were set as 100% (*n* = 3). (**D**) The protein expression of TRAPα, TRAPβ, TRAPγ, and TRAPδ from WT islets treated with 2.8 mM and 16.7 mM for 4 hours was examined by Western blots (*n* = 3). (**E**) Quantification of TRAPα, TRAPβ, TRAPγ, and TRAPδ protein levels in **D**. (**F**) The mRNA levels of TRAPα, TRAPβ, TRAPγ, and TRAPδ were measured in islets isolated from 8- to 12-week-old control (Con) and TRAPα-βKO mice. The mRNA levels in Con islets were set as 100% (*n* = 4). (**G**) The protein expression of TRAPα, TRAPβ, TRAPγ, and TRAPδ from 8- to 12-week-old Con and TRAPα-βKO mice was examined by Western blots (*n* = 4–12). (**H**) Quantification of TRAPα, TRAPβ, TRAPγ, and TRAPδ protein levels in **G**. Values are reported as mean ± SEM. *P < 0.05, **P < 0.01, ***P < 0.001, and ****P < 0.0001, unpaired Student’s *t* test and 2-way ANOVA.

By immunofluorescence in both human and mouse pancreas, we found that TRAPα was highly expressed in pancreatic islets compared with exocrine pancreatic tissue. Notably, TRAPα colocalized primarily with INS rather than glucagon (GCG) or somatostatin, suggesting that TRAPα is more highly expressed in cells than islet cell types (Figure 1, A and B). To further investigate the subcellular localization of TRAPα, we performed confocal microscopy analyses using antibodies against TRAPα, KDEL (an ER marker), or TGN46 (a trans-Golgi network marker). Consistent with our previous findings in pancreatic cell lines (26), TRAPα exhibited strong colocalization with KDEL, indicating its predominant localization in the ER (Supplemental Figure 1; supplemental material available online with this article; https://doi.org/10.1172/JCI179845DS1).

Glucose-stimulated INS biosynthesis and secretion are characteristic features of cells. To determine whether the expression of TRAPα responds to glucose, we treated isolated mouse islets with low (2.8 mmol/L) and high (16.7 mmol/L) glucose concentrations for 4 hours and found that TRAPα as well as other subunits of TRAP complexes were all upregulated at both transcriptional and translational levels (Figure 1, C–E). Among all 4 subunits, TRAPα was the most upregulated mRNA(Figure 1C).

To further determine the role of TRAPα in cells, we established a TRAPα-βKO mouse line (Supplemental Figure 2A) using homologous recombination mediated by *Ins2*-IRES-Cre, in which an internal ribosome entry site after the *Ins2* stop codon but before the poly-A sequence allows for Cre expression and activity restricted to β cells without detectable expression in the hypothalamus and without altering the expression of *Ins2* or *Ins1*, circulating INS, or blood glucose in either the fasting state or during intraperitoneal glucose tolerance tests (IPGTTs) at age 3–4 months (30). To further confirm these findings, we set up 2 additional mouse cohorts including male *Ins2*-IRES-Cre mice and WT control mice. One cohort of mice had been fed a chow diet for up to 4 months, and another cohort of mice had been fed a chow diet for 2 months followed by additional 2-month HFD. Again, no differences were observed in body weight and glucose tolerance between *Ins2*-IRES-Cre and WT mice fed the chow diet (Supplemental Figure 3, A–C) or fed the HFD (Supplemental Figure 3, D and E).

Next, we examined efficiency of TRAPα deletion in TRAPα-βKO mice. A markedly decreased expression of TRAPα was independently confirmed by qRT-PCR (Figure 1F), Western blot (Supplemental Figure 2, B and C), and immunohistochemistry (Supplemental Figure 2D). The percentage of TRAPα-positive cells decreased 79% in TRAPα-βKO islets compared with that of the control (Supplemental Figure 2, E and F). Interestingly, although 3 other subunits (TRAPβ, TRAPγ, and TRAPδ) of the TRAP complex were upregulated at the mRNA level in TRAPα-βKO islets (Figure 1F), TRAPα deficiency resulted in decreased protein levels for all 3 other subunits (Figure 1, G and H), suggesting that TRAPα is important for the stability (or translational efficiency) of other subunits of the TRAP complex.

**Figure 2:**
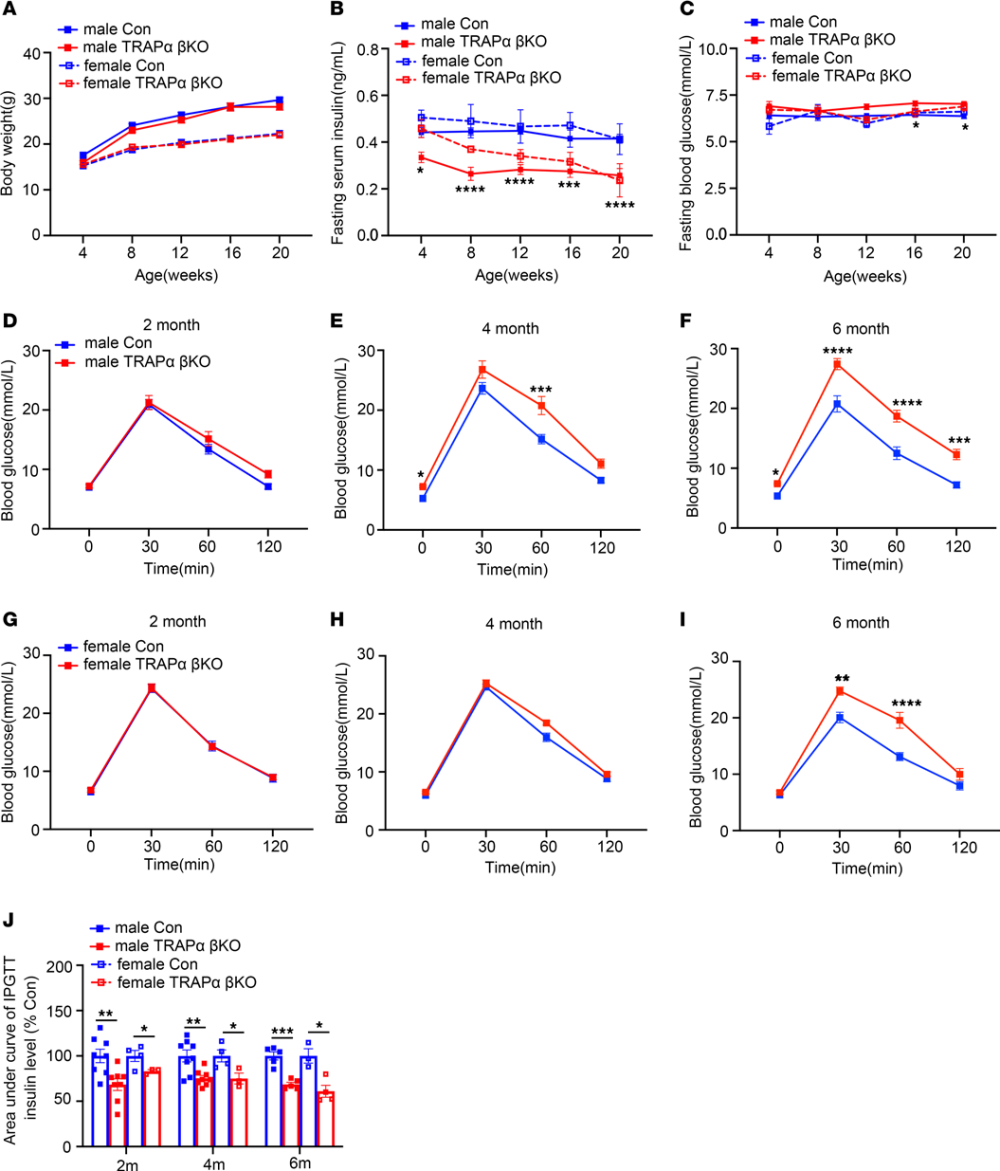
TRAPα-βKO decreases circulating INS and causes glucose intolerance. (**A**) The body weight of male and female control (Con) and TRAPα-βKO mice (*n* = 10). (**B**) Fasting serum INS levels of the same groups of mice as in **A**. (**C**) Fasting blood glucose levels of the same groups of mice as in **A**. (**D**–**F**) IPGTTs were performed in 2-, 4-, and 6-month-old Con and TRAPα-βKO male mice (Con, *n* = 5–10; TRAPα-βKO, *n* = 5–8). (**G**–**I**) IPGTTs were performed in 2-, 4-, and 6-month-old Con and TRAPα-βKO female mice (Con, *n* = 6-7; TRAPα-βKO, *n* = 8-10). (**J**) The AUC for INS level of the same groups of mice as **D**–**I**. Values are reported as mean ± SEM. *P < 0.05, **P < 0.01, ***P < 0.001, and ****P < 0.0001, unpaired Student’s *t* test and 2-way ANOVA.

Next, we examined the impact of β cell TRAPα deficiency on blood glucose homeostasis. TRAPα-βKO mice started to have decreased circulating INS as early as 1 month of age without affecting body weight (Figure 2, A and B). Starting from 4 months of age, TRAPα-βKO male mice progressively developed INS-deficient impaired glucose intolerance (IGT), which became more profound at 6 months of age (Figure 2, D–F, and Supplemental Figure 4, A–J). Female TRAPα-βKO mice exhibited milder phenotypes with delayed onset of IGT (Figure 2, G–J, and Supplemental Figure 4, K–M).

### TRAPα-βKO impairs PPI translocation and decreases INS production but does not affect glucose-stimulated INS secretory responsiveness.

**Figure 3:**
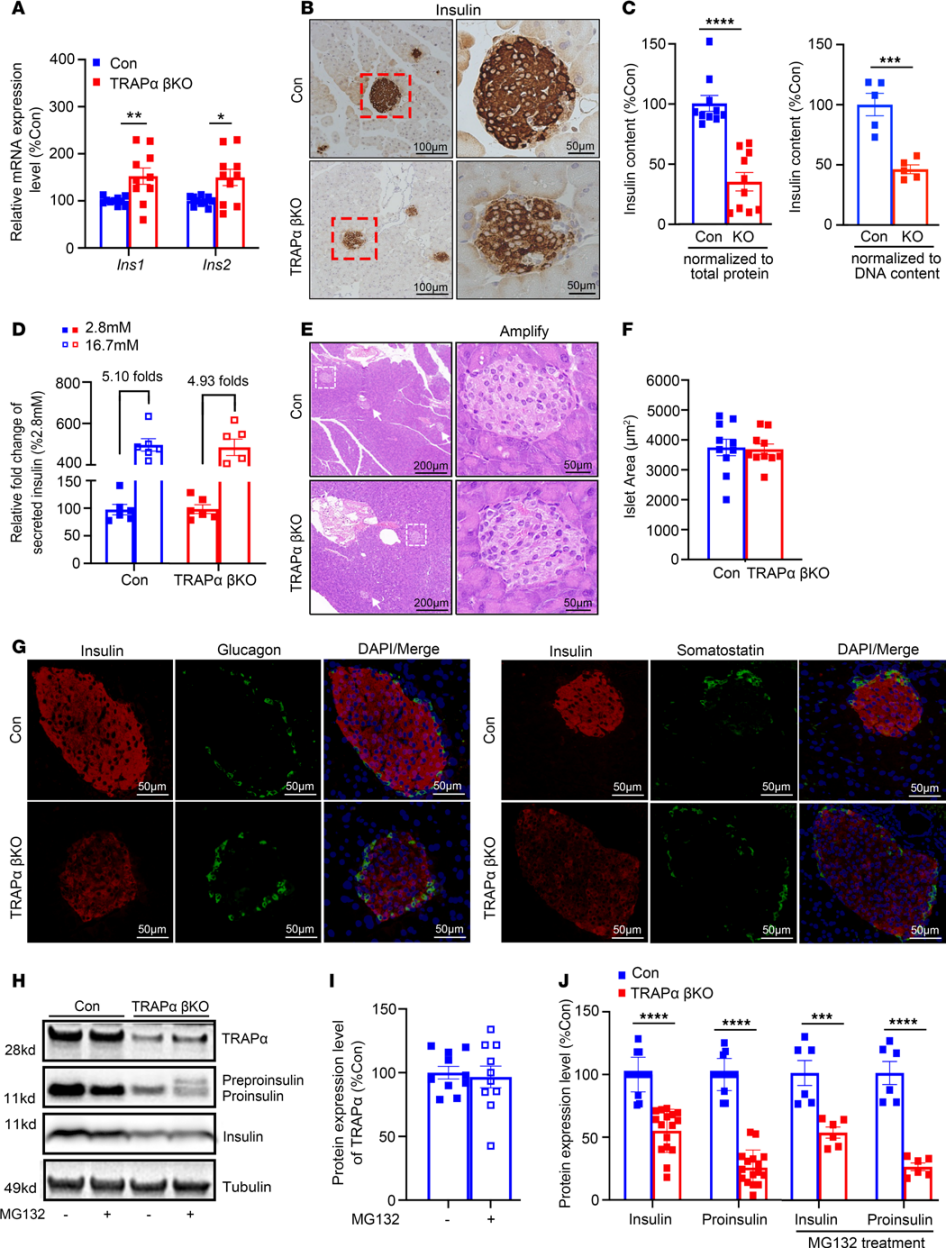
TRAPα-βKO impairs PPI translocation and decreases INS production but does not affect GSIS or islet size and cell composition. (**A**) mRNA levels of INS genes, including *Ins1* and *Ins2* from 8- to 12-week-old male control (Con) and TRAPα-βKO mice islets (*n* = 10). (**B**) Immunohistochemistry staining was performed using anti-INS in pancreatic sections of 8- to 12-week-old male Con and TRAPα-βKO mice. (**C**) INS content from 8- to 12-week-old male Con and TRAPα-βKO mouse islets was measured using INS ELISA normalized with either islet total protein (left) or islet DNA content (right) to minimize the effects of interislet heterogeneity. The INS content from Con mouse islets was set as 100% (*n* = 5–10). (**D**) GSIS was performed using islets isolated from 8- to 12-week-old male Con and TRAPα-βKO mice (*n* = 6). Secreted INS from male control islets treated with 2.8 mM glucose was set as 100% (*n* = 6 in each group). (**E**) H&E staining was performed of pancreases of 8- to 12-week-old male Con and TRAPα-βKO mice. (**F**) The quantification of the islets size of **E** (*n* = 10 in each group). (**G**) Pancreatic sections of 8- to 12-week-old male Con and TRAPα-βKO mice were immunostained with anti-INS (red), anti-GCG (green, left panel), or anti-somatostatin (green, right panel) as indicated. (**H**) Islets isolated from 8- to 12-week-old male Con and TRAPα-βKO mice were treated with or without MG132 (10 μM) for 2 hours before being analyzed by Western blotting using anti-INS or anti-TRAPα, as indicated. (**I**) Quantification of TRAPα protein levels in islets treated with or without MG132 in **H** (*n* = 10). (**J**) Quantification of PI and INS in TRAPα-βKO islets with and without MG132 treatment shown in **H** (*n* = 6–16). Values are reported as mean ± SEM. *P < 0.05, **P < 0.01, ***P < 0.001, ****P < 0.0001, unpaired Student’s *t* test and 2-way ANOVA.

Decreased circulating INS (Figure 2, B and J) could be caused by a decrease in INS gene expression a decrease of INS secretory responsiveness, a decrease of islet β cell mass, or a decrease of INS content due to defective INS biosynthesis. We found that the mRNA levels of both *Ins1* and *Ins2* were not decreased; surprisingly, they were upregulated in TRAPα-βKO islets (Figure 3A). Additionally in isolated islets, we examined glucose-stimulated INS secretion (GSIS; with low [2.8 mmol/L] and high [16.7 mmol/L] glucose concentrations) and found that although the absolute amount of INS was markedly decreased, demonstrated both by immunohistochemistry with anti-INS (Figure 3B) and by ELISA for INS measurement (Figure 3C), the fold-change of stimulated INS secretion was not affected in TRAPα-βKO islets (Figure 3D), demonstrating that TRAPα deficiency does not affect glucose-stimulated secretory responsiveness. Furthermore, neither islet size (Figure 3, E and F) nor the composition and distribution of 3 major islet cell types (α, β, and δ cells) (Figure 3G) were significantly affected by TRAPα-βKO. These data suggest the reduction in circulating INS was not caused by decreased INS gene transcription or β cell mass but might result from reduced INS content that was likely caused by defective INS biosynthesis.

To dissect which steps of INS biosynthesis might be affected by TRAPα, we first examined PPI translocation into the ER via the Sec61 translocon (1). The half-life of PPI is normally only 2–3 minutes, in part because untranslocated PPI is rapidly degraded by the proteasome (31). To detect untranslocated PPI, we treated isolated islets with or without MG132 (a proteasomal inhibitor that prevent untranslocated PPI from degradation; ref. 12) before lysis. We found that, without affecting TRAPα expression (Figure 3, H and I), MG132 treatment allowed PPI to become visible in TRAPα-βKO islets (Figure 3H, lane 2 vs. lane 4). TRAPα-βKO was accompanied by markedly decreased PI and mature INS (Figure 3, H and J), suggesting that TRAPα deficiency impairs PPI translocation and INS biosynthesis.

### TRAPα is important for β cell ER function, and its deficiency disturbs PI oxidative folding in the ER, inducing ER stress.

**Figure 4:**
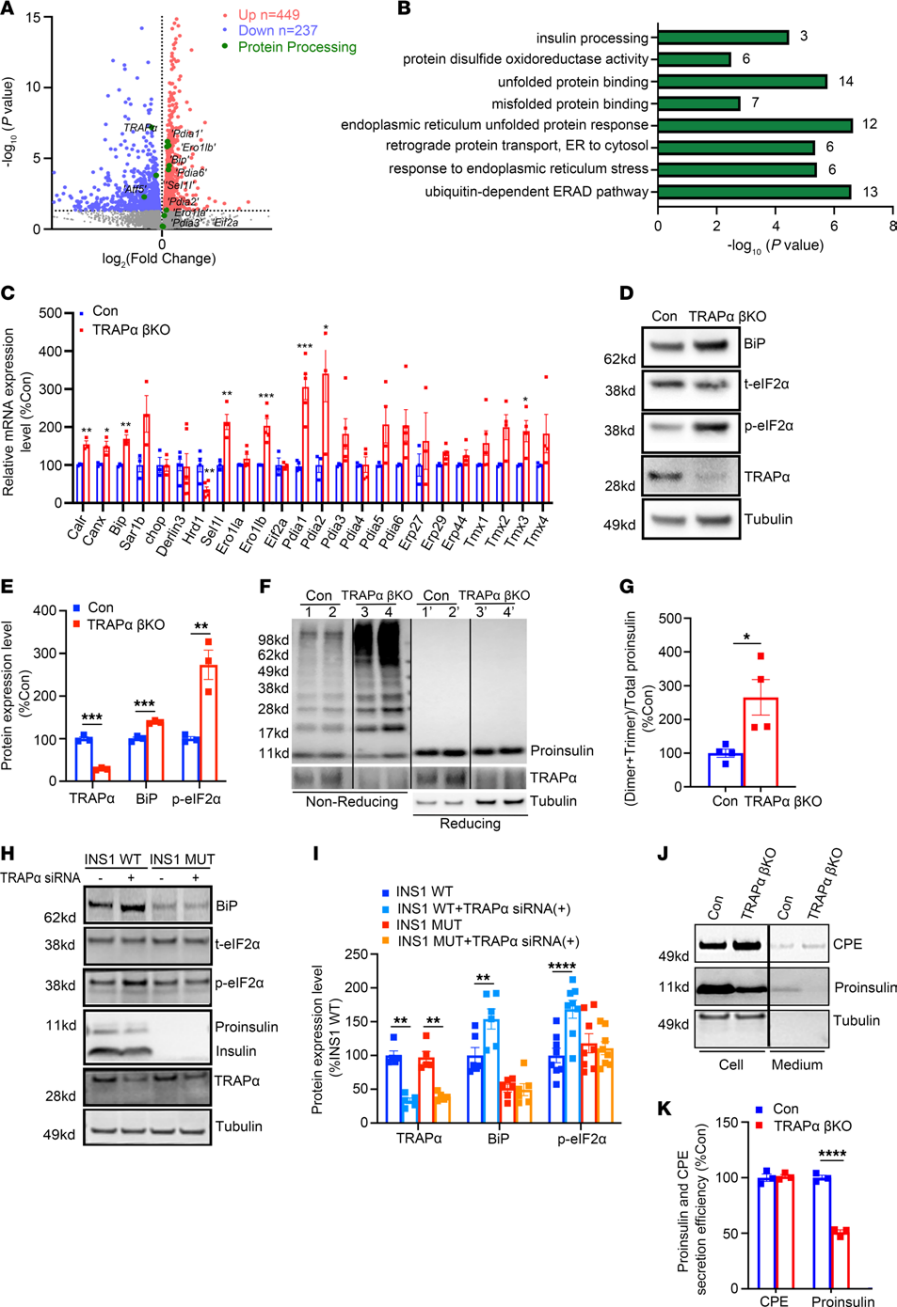
TRAPα is important for β cell ER function, and its deficiency causes PI misfolding and ER stress. (**A**) Transcriptome analyses using islets isolated from 8-week-old control (Con) and TRAPα-βKO male mice (*n* = 3 in each group). (**B**) Gene Ontology analyses of RNA-Seq data showed the pathways associated with ER genes that were significantly upregulated in islets of 8-week-old TRAPα-βKO male mice (as in **A**). (**C**) mRNA levels of indicated genes were measured by real-time quantitative PCR (qRT-PCR) in islets from 8- to 12-week-old Con and TRAPα-βKO male mice islets (*n* = 3-6). (**D**) The protein expression of TRAPα, BiP, and p-eIF2α from 12-week-old Con and TRAPα-βKO male mice was examined by Western blots. (**E**) Quantification of TRAPα, BiP, and p-eIF2α protein levels in **D** (*n* = 3 in each group). (**F**) The oxidative folding of PI in islets from 8- to 12-week-old Con and TRAPα-βKO male mice was analyzed by Western blots under both nonreducing and reducing conditions. (**G**) The amounts of PI dimers plus trimers under nonreducing conditions compared with total PI under reducing conditions were quantified and calculated. The ratios of dimers plus trimers to total PI in control islets were set to 100% (*n* = 4). (**H**) INS1 control cells (WT) and INS1 with both *Ins1* and *Ins2* gene-deleted cells (Mut) were transfected with either TRAPα siRNA or scrambled siRNA. At 72 hours after transfection, the cells were lysed, and ER stress responses were analyzed by Western blots, as indicated. (**I**) Quantification of **H** from 5–8 independent experiments. (**J**) Isolated islets were incubated in RPMI 1640 without FBS for 6 hours; the secretion of PI and CPE was analyzed by Western blots. (**K**) PI and CPE in the islets and media were quantified from **J** in 3 independent experiments. The secretion efficiency of PI and CPE was calculated, and secretion efficiency of PI and CPE in control islets was set to 100%. Values are reported as mean ± SEM. *P < 0.05, **P < 0.01, ***P < 0.001, and ****P < 0.0001, unpaired Student’s *t* test and 2-way ANOVA.

The topology of the TRAPα protein is such that most of the protein is present on the luminal side of the ER membrane (32), suggesting that, in addition to its role in PPI translocation, TRAPα has a function within the ER lumen. Interestingly, transcriptome analyses revealed that the expression of 449 genes was upregulated and that of 237 genes was downregulated in TRAPα-βKO islets (Figure 4A). The upregulated ER genes were related to unfolded or misfolded protein binding, responses, and degradation, as well as protein disulfide formation and isomerization (Figure 4B). qRT-PCR confirmed that the master ER stress regulator binding-immunoglobulin protein (*Bip*), the major ER oxidoreductases *Ero1lb* and protein disulfide isomerase, and the key ER chaperone involved in ER-associated protein degradation, *Sel1l*, were all upregulated, whereas the mRNA encoding *Hrd1* (an E3 ubiquitin ligase) was decreased (Figure 4C). Furthermore, Western blotting confirmed that both BiP and phosphorylated eukaryotic translation initiation factor 2 subunit 1 (p-eIF2α) were increased in TRAPα-βKO islets (Figure 4D, quantified in Figure 4E). These multiple independent approaches indicate that TRAPα-βKO induces ER stress and activates the unfolded protein response (UPR).

Next, we asked what factor(s) mediate the ER stress induced by TRAPα deficiency. PI is the most abundant protein in β cell ER, and its oxidative folding is sensitive to impairment of ER function (17, 21). To examine the possible role of TRAPα in PI folding in the ER, we examined oxidative folding of PI under both nonreducing and reducing conditions, using established methods (14, 20). When normalized for comparable levels of total PI (detected under reducing conditions, as seen in Figure 4F, for which total islet protein was intentionally increased), the number of higher molecular weight, disulfide-linked, PI-containing protein complexes was dramatically increased, and expression of monomeric PI was decreased in TRAPα-βKO islets (Figure 4F, quantified in Figure 4G). These data suggest TRAPα-βKO impairs PI oxidative folding and promotes PI misfolding in the ER.

To determine whether misfolded PI is the direct cause of ER stress in TRAPα deficiency cells, we established a rat insulinoma INS1 cell line in which *Ins1* plus *Ins2* were knocked out. As shown in Figure 4, H and I, downregulating expression of TRAPα with siRNA in control INS1 cells increased expression of BiP and p-eIF2α that was consistent with the results found in TRAPα-βKO islets (Figure 4, D and E). However, in INS1 cells lacking PI expression, TRAPα deficiency did not activate UPR (Figure 4, H and I), indicating that ER stress caused by TRAPα-βKO is likely mediated by an increase of misfolded PI. Interestingly, although the PI secretion rate was decreased due to PI misfolding, TRAPα-βKO did not affect secretion of carboxypeptidase E (CPE) (Figure 4, J and K, and Supplemental Figure 6), suggesting that ER export is still functional in TRAPα-βKO islets and TRAPα has a selective role in PI folding and secretion. Together, these data demonstrate that TRAPα is critical not only for PPI translocation across the ER membrane but also for PI folding and export from the ER. TRAPα deficiency causes PI misfolding, ER stress, and increased phosphorylation of eIF2α, which all contribute to decreased INS production.

### TRAPα-βKO weakens the compensatory ability of β cells to respond to HFD, aggravating HFD-induced β cell dysfunction and diabetes.

**Figure 5:**
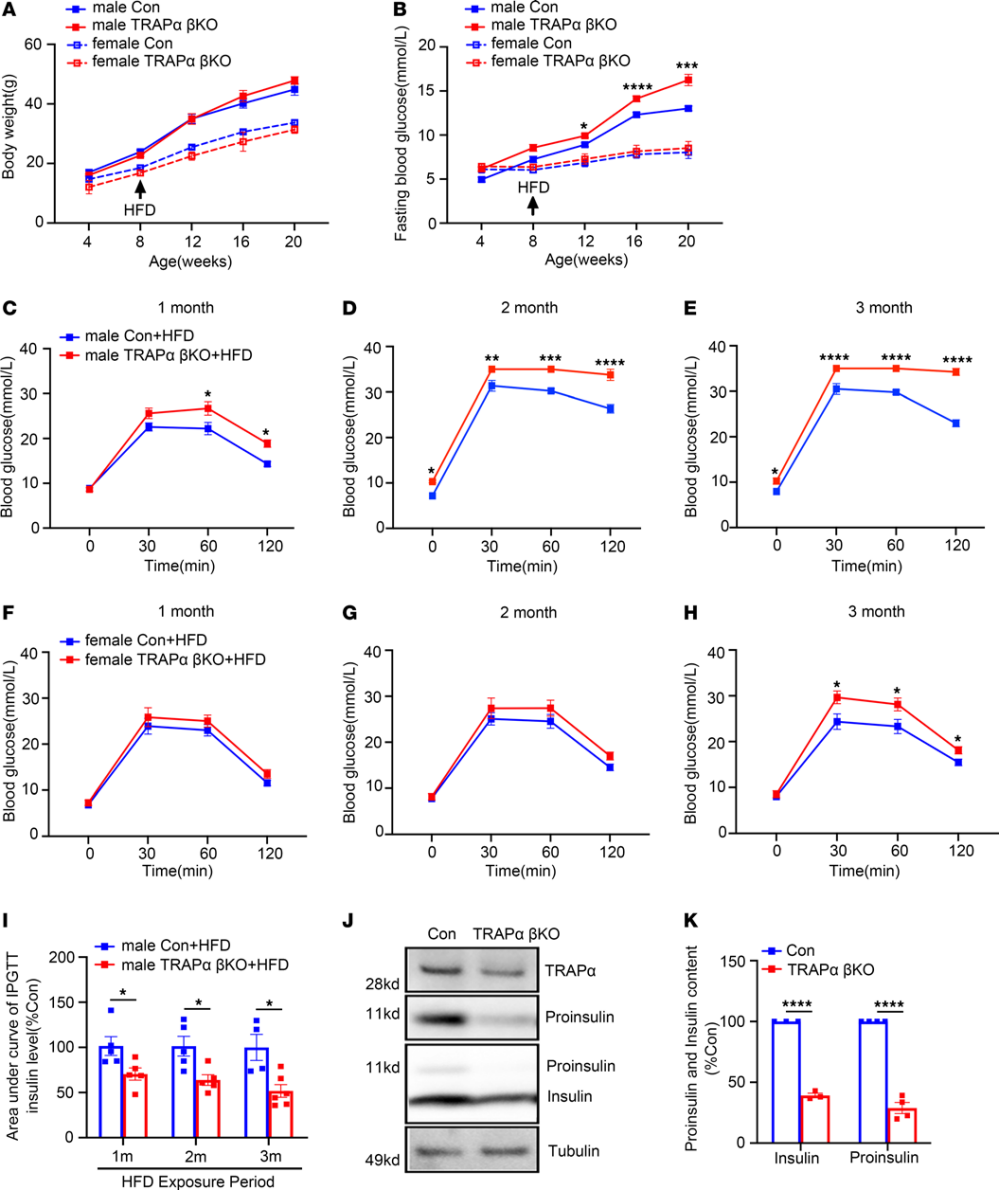
TRAPα-βKO weakens compensative ability of β cells to an HFD. (**A**) Control (Con) and TRAPα-βKO male and female mice (8 weeks old) were fed an HFD for 12 weeks. Body weight was measured every 4 weeks during the 20-week period (*n* = 5–8 male mice, *n* = 4–6 female mice). (**B**) Fasting blood glucose levels of the same groups of mice as in **A** were measured monthly. (**C**–**E**) IPGTTs in Con and TRAPα-βKO male mice after 1–3 months of HFD feeding (*n* = 4–8). (**F**–**H**) IPGTTs in Con and TRAPα-βKO female mice after 1–3 months of HFD feeding (*n* = 5). (**I**) The AUC for INS levels of the same group of male mice as shown in **C**–**E**. The raw non-normalized INS levels during IPGTTs are shown in Supplemental Figure 7, A–C, for male mice and Supplemental Figure 7D for female mice. (**J**) Islets isolated from 12-week HFD-fed Con and TRAPα-βKO male mice were lysed. PI and INS were analyzed by Western blots. (**K**) Quantification of PI and INS in **J** (*n* = 3–4). Values are reported as mean ± SEM. *P < 0.05, **P < 0.01, ***P < 0.001, and ****P < 0.0001, unpaired Student’s *t* test and 2-way ANOVA.

Compensatory increases of INS biosynthesis and secretion are features of patients with prediabetes. Because TRAPα is critical for INS biosynthesis, we asked whether TRAPα-βKO could impair β cell compensatory response to dietary challenge. We fed mice an HFD beginning at 8 weeks of age and found that, with comparable weight gain in both male and female mice (Figure 5A), HFD feeding resulted in elevated fasting blood glucose levels after 12 weeks in TRAPα-βKO male mice. However, no significant changes in fasting blood glucose were observed after 20 weeks in TRAPα-βKO female mice fed the HFD (Figure 5B). IPGTTs also supported the notion that TRAPα-βKO exacerbated IGT as early as 1 month after HFD feeding in male mice (Figure 5, C–E), whereas in female mice, IGT was observed only after 3 months of HFD feeding (Figure 5, F–H). Furthermore, in TRAPα-βKO mice, circulating INS during IPGTT in both male and female mice (Figure 5I and Supplemental Figure 7), as well as islet PI and INS content of male mice, were reduced (Figure 5, J and K, and Supplemental Figure 5, G–K), suggesting that TRAPα-βKO weakens β cell compensatory ability in response to an HFD, which was more prominent in male than female mice.

**Figure 6:**
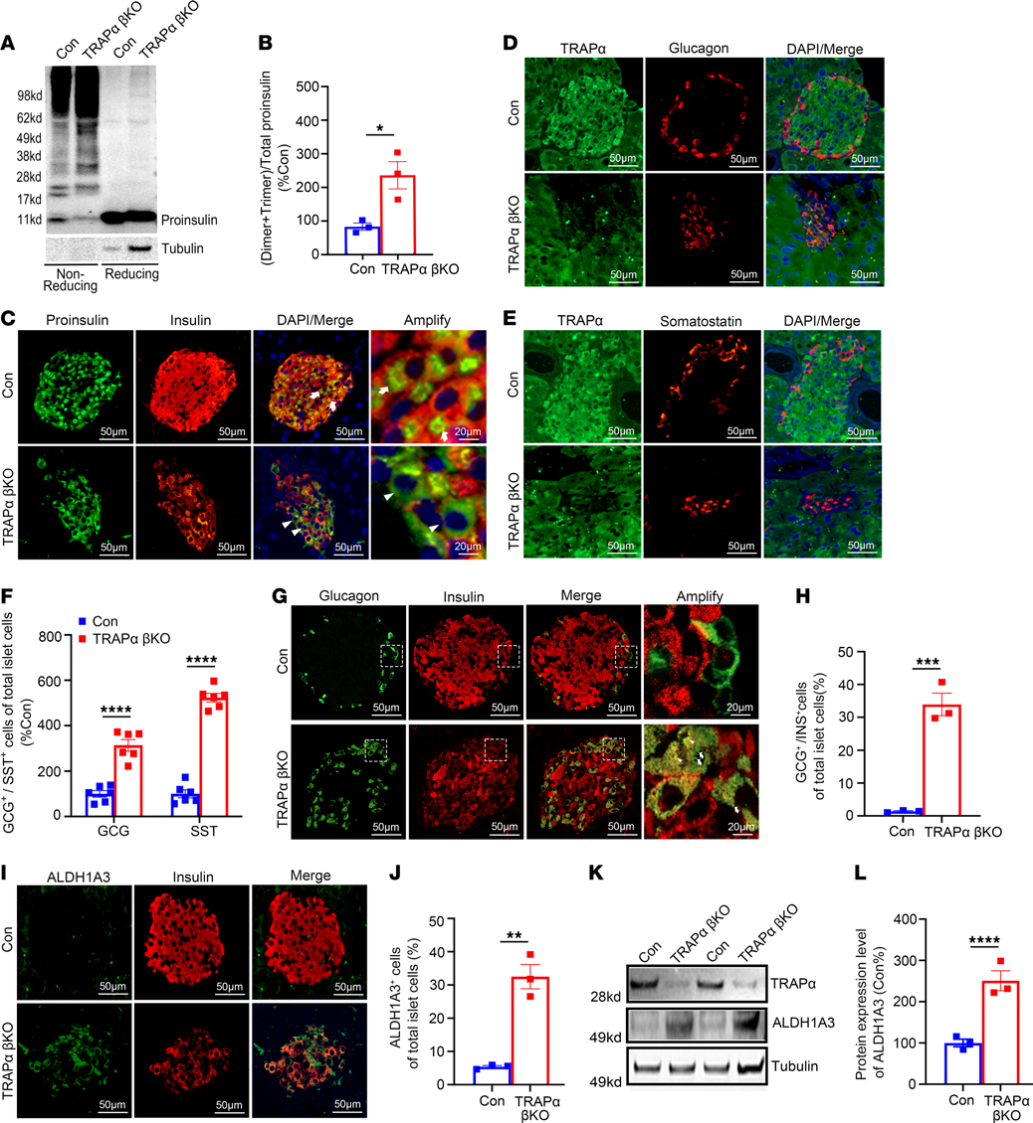
TRAPα-βKO causes PI misfolding, alters islet cell composition, and increases ALDH1A3 expression in male mice under HFD feeding. (**A**) The oxidative folding of PI in islets from 3-month HFD control (Con) and TRAPα-βKO male mice was analyzed using Western blots under both nonreducing and reducing conditions. (**B**) The amounts of PI dimers plus trimers under nonreducing conditions compared with total PI under reducing conditions were quantified and calculated. The ratios of dimers plus trimers to total PI in control islets were set to 100% (*n* = 3). (**C**) Pancreatic sections from Con and TRAPα-βKO male mice fed a chow diet for 2 months followed by additional 3 months of HFD feeding were immunostained with anti-PI (green) and anti-INS (red). (**D** and **E**) Representative immunofluorescence images are shown, displaying the staining of anti-somatostatin (red) or anti-GCG (red) along with anti-TRAPα (green) in pancreatic sections from Con and TRAPα-βKO male mice fed an HFD, as in **C**. (**F**) The percentages of GCG-positive (α cells) or somatostatin-positive (δ cells) cells in total islet cells shown in **D** and **E** were quantified and calculated (*n* = 6). (**G**) Confocal analysis of pancreatic sections costained with anti-GCG (green) and anti-INS (red) in Con and TRAPα-βKO male mice fed an HFD, as in **C**. (**H**) Percentages of GCG^+^/INS^+^ cells in total islet cells shown in **G**. (**I**) Co-immunostaining for ALDH1A3 (green) and INS (red) in pancreatic sections of Con and TRAPα-βKO male mice fed an HFD (as in **C**). (**J**) Percentages of ALDH1A3^+^ cells in total islet cells shown in **I**. (**K**) Western blot examining the expression of ALDH1A3 in islets of Con and TRAPα-βKO male mice fed an HFD, as in **C**. (**L**) Quantification of ALDH1A3 from Western blot shown in **K** (*n* = 3). The expression of ALDH1A3 in control islets was set as 100%. Values are reported as mean ± SEM. *P < 0.05, **P < 0.01, ***P < 0.001, and ****P < 0.0001, unpaired Student’s *t* test and 2-way ANOVA.

To further examine β cell function in TRAPα-βKO mice fed an HFD, we examined PI folding in the islets of HFD-fed mice and again observed an increase of misfolded PI with diminished monomeric PI (Figure 6, A and B). By indirect immunofluorescence experiments, we observed that almost all β cells of control islets expressed both PI and INS, and PI was mostly concentrated in a juxtanuclear Golgi compartment (Figure 6C, arrows). However, in TRAPα-βKO islets, we observed heterogeneity with PI-positive, INS-negative β cells, and PI was more diffusely distributed throughout the cytoplasm (Figure 6C, arrowheads), suggesting that misfolded PI was retained in the ER in cells that contained little mature INS. To determine whether β cell loss was associated with apoptosis, we performed TUNEL staining and found that although TRAPα deficiency did not increase β cell apoptosis in mice fed the chow diet (Supplemental Figure 8, A and B), it caused significantly increased β cell death in TRAPα-βKO mice fed the HFD (Supplemental Figure 8, C and D). Furthermore, unlike chow-fed mice (Figure 3G), HFD significantly increased the percentages of GCG-positive cells and somatostatin-positive cells (Figure 6, D–F) in TRAPα-βKO mice. Moreover, in HFD-fed TRAPα-βKO male mice, the number of islet cells coexpressing INS and GCG increased (Figure 6, G and H). Furthermore, both Western blot and confocal analysis showed that ALDH1A3, a marker of β cell dedifferentiation (33), was upregulated in islets of TRAPα-βKO mice fed the HFD (Figure 6, I–L). Together, these data indicate that TRAPα is important for compensatory potential of β cells, and TRAPα deficiency sensitizes β cells to dietary stress, leading to β cell dysfunction, apoptosis, and diabetes.

### TRAPα expression decreases in islets of HFD-fed male mice and islets of patients with T2D; decreased TRAPα expression correlates with less INS content in human islets.

**Figure 7:**
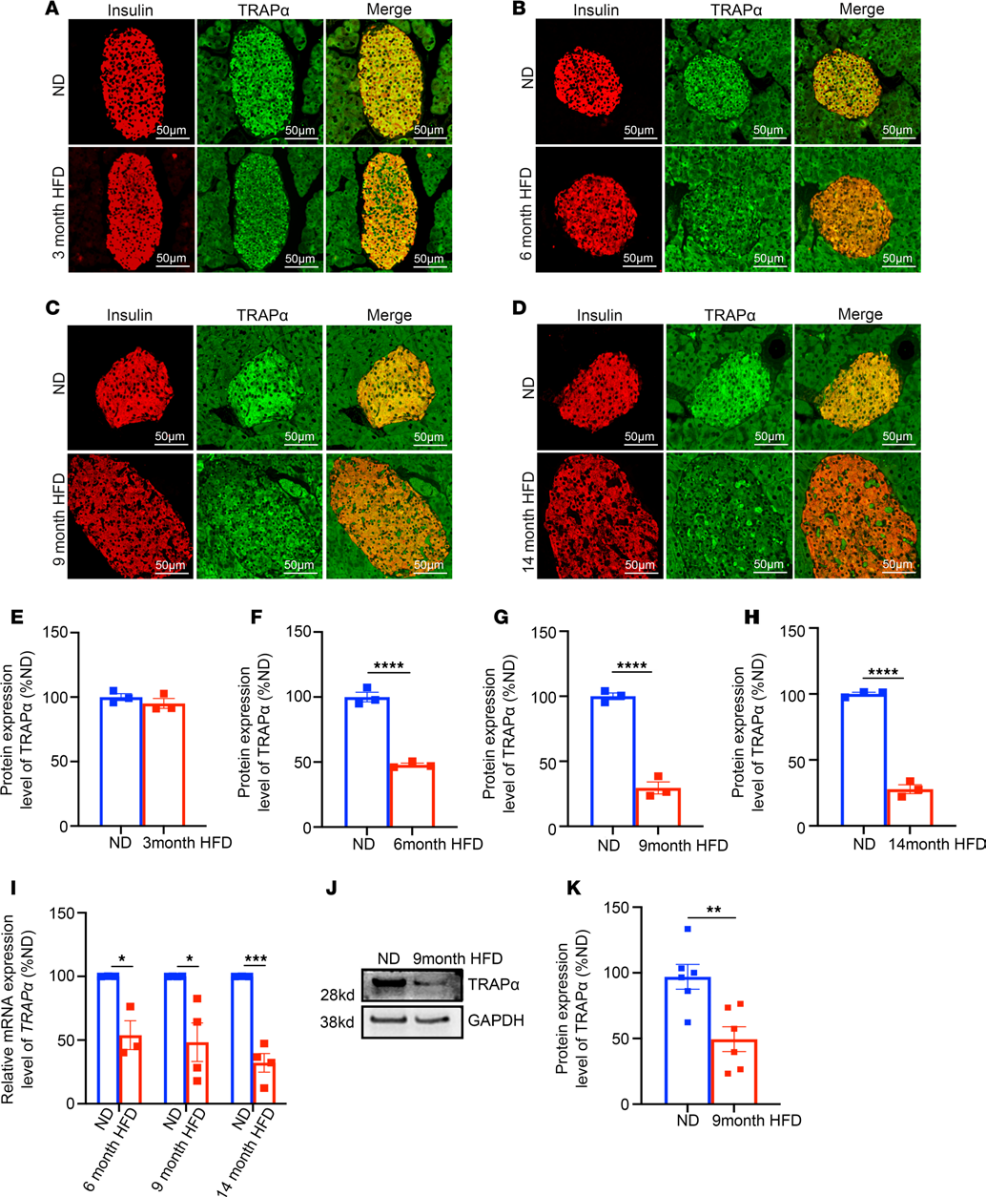
An HFD downregulates TRAPα expression in mouse islets. (**A**–**D**) WT male mice (2 months old) were fed an HFD for up to 14 months. The expression of TRAPα was examined by immunofluorescence with anti-TRAPα (green) at 3, 6, 9, and 14 months of an HFD. The expression of TRAPα in mice fed a normal diet (ND) was examined at the same ages as that of HFD mice. (**E**–**H**) Quantification of TRAPα and INS protein levels shown in **A**–**D** (*n* = 3). (**I**) The mRNA levels of TRAPα were measured by real-time quantitative PCR PCR (qRT-PCR) in islets isolated from male mice fed an HFD for 6, 9, and 14 months and same-age male mice fed an ND. The mRNA levels in islets of mice fed an ND were set as 100% (*n* = 3–4). (**J**) Protein levels of TRAPα at 9 months of HFD feeding were examined by Western blots. (**K**) Quantification of protein levels of TRAPα shown in **J** (*n* = 6). Values are reported as mean ± SEM. *P < 0.05, **P < 0.01, ***P < 0.001, and ****P < 0.0001, unpaired Student’s *t* test and 2-way ANOVA.

GWAS link SNPs of TRAPα to glycemic phenotypes in humans (28, 29). We asked whether there was an alteration of TRAPα expression in β cells under conditions leading to the development of T2D. Using multiple approaches, we first examined TRAPα expression at both the mRNA and protein levels in WT mice that had been fed the HFD for up to 14 months. We found that TRAPα expression was progressively decreased in pancreatic islets but not in pancreatic exocrine tissue (Figure 7, A–K).

**Figure 8:**
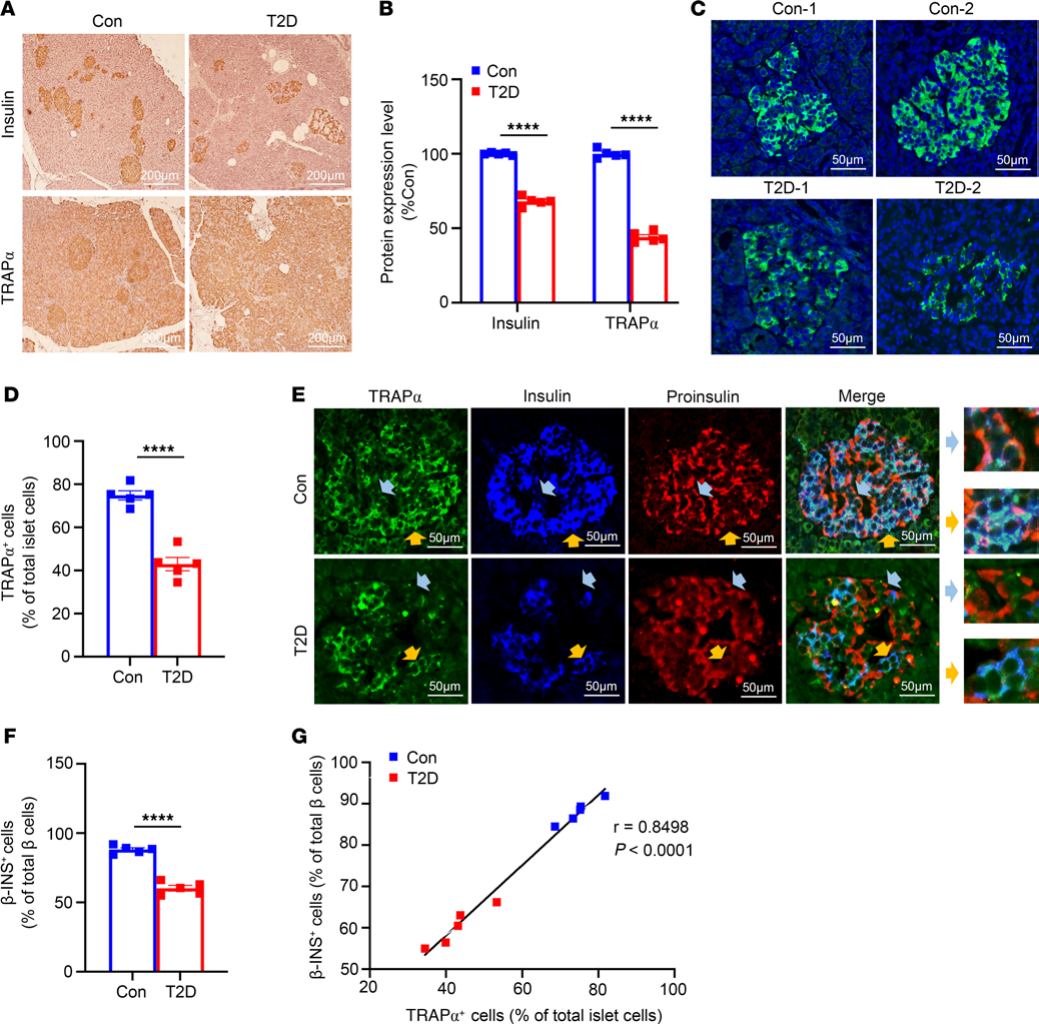
Decreased expression of TRAPα is evident in islets of patients with T2D and is correlated with decreased mature INS. (**A**) Pancreatic sections from donors without (Con) or with T2D were stained for INS (top) and TRAPα (bottom). (**B**) Quantification of TRAPα and INS in **A** (*n* = 10 donors, 3 sections/donor). (**C**) TRAPα (green) expression in Con and T2D islets; nuclei stained with DAPI (blue). (**D**) Percentage of TRAPα^+^ cells among total islet cells. (**E**) Costaining for TRAPα (green), INS (blue), and PI (red) shows 2 β cell subtypes: β-PI^+^ (TRAPα^–^INS^–^PI^+^, blue arrows) and β-INS^+^ (TRAPα^+^INS^+^PI^–^, orange arrows). (**F**) Proportion of β-INS^+^ cells among all β cells (β-INS^+^ + β-PI^+^) in Con and T2D islets. (**G**) Correlation between percentage TRAPα^+^ islet cells (**D**) and percentage β-INS^+^ cells (**F**) per islet. Values are mean ± SEM. ****P < 0.0001, unpaired Student’s *t* test and 2-way ANOVA.

Next, we examined the expression of TRAPα in pancreatic sections of patients with T2D and found that TRAPα expression in islets (Figure 8A) decreased by more than 50% (Figure 8B), and the percentage of TRAPα-positive cells inside islets was also decreased (Figure 8, C and D). Because TRAPα was mostly expressed in β cells (Figure 1A), the decrease of TRAPα in T2D islets may have been due to a reduction of β cells. To take a closer look at this, we costained pancreatic sections with anti-TRAPα, anti-PI, and anti-INS. As we have previously demonstrated (1, 18) and verify in Figure 8E, there were subpopulations of β cells with different relative levels of PI, INS, and TRAPα. One subpopulation of β cells exhibited persistent PI staining but diminished staining of INS and TRAPα (which we refer to as β-PI^+^; Figure 8E, light blue arrows), which might signify defective INS maturation (18) in β cells with diminished TRAP. Another subpopulation of β cells was strongly positive for both INS and TRAPα (β-INS^+^; Figure 8E, orange arrows). Interestingly, these cells were weakly positive for PI, suggesting efficient PI export and processing to INS. Importantly, the percentage of β-INS^+^ cells as a fraction of all β cells (β-PI^+^ plus β-INS^+^) declined in the T2D islets (Figure 8F). Notably, the percentage of β-INS^+^ cells in islets positively correlated with TRAPα^+^ cells in islets in both T2D and control groups (Figure 8G). Together, these data indicate decreased TRAP in an abnormally enlarged subpopulation of islet β cells that match the β cells with loss of INS in T2D, suggesting the hypothesis that TRAPα deficiency could contribute to INS deficiency in these failing β cells.

## Discussion

Our previous study demonstrated that deletion of TRAPα in pancreatic β cell lines impairs PPI translocation and INS production in vitro (26). However, to date, no direct evidence shows the pathophysiological role of TRAPα in pancreatic β cells of whole animals. To our knowledge, the present work is the first designed to understand the role of TRAPα/SSR1 in β cell physiology in vivo and the potential impact of its deficiency as a contributor to a predisposition to diet-induced diabetes and T2D. Our data reveal that TRAPα-βKO not only impedes PPI translocation across the ER membrane (Figure 3H) but also impairs PI oxidative folding in the ER (Figure 4, F and G), resulting in a noteworthy reduction in islet INS and circulating INS (Figure 2J; Figure 3, B and C; and Supplemental Figure 5). Transcriptome analyses (confirmed by qRT-PCR) revealed activation of UPR upon loss of TRAPα expression in islets (Figure 4, C–E). Importantly, the UPR phenotype is dependent upon INS gene expression in INS1 cells with decreased TRAPα expression (Figure 4, H and I). The data reveal an unexpected role of TRAPα in PI oxidative folding in the ER and support a notion that misfolded PI caused by TRAPα deficiency is a direct trigger of ER stress. Additionally, our observations indicate that TRAPα deficiency led to IGT influenced both by age and diet (Figures 2 and 5). These data highlight that TRAPα may participate in the intricate interplay between genetic predisposition and environmental factors.

Given the important role of TRAPα in regulating INS biosynthesis and β cell ER function, it would be important to determine possible alterations of TRAPα expression in islets of patients with T2D. Bugliani et al. (34) performed microarray analysis in human T2D islets and reported a 3.5-fold decrease in TRAPα mRNA expression in comparison with nondiabetic islets. These findings were consistent with data from the National Center for Biotechnology (NCBI), which showed a downregulation of TRAPα mRNA in T2D islets (acccession GSE25724) (35). However, the reduction of TRAPα expression was not confirmed by 2 studies in which the trends of decreased TRAPα mRNA were noted without reaching statistical significance (36, 37). Therefore, the magnitude of the changes in TRAPα mRNA during the development and progression of T2D remains to be determined. However, our current studies reveal that TRAPα protein expression level, indeed, decreased in islets of patients with T2D (Figure 8), and both mRNA and protein levels declined in islets of mice fed chronically with HFD (Figure 7).

An intriguing question arising from our study is whether the decreased TRAPα expression contributes to the failure of β cell compensation in T2D. Compensatory mechanisms involve a series of adaptive responses aimed at increasing capacity of β cells in the face of increasing metabolic demands. These mechanisms are essential for preventing the development of diabetes and mitigating its progression (1, 38–40). In the present study. we observed impaired PI oxidative folding in the ER of TRAPα-deficient β cells, resulting in an accumulation of misfolded PI and intolerance of glucose under HFD conditions (Figure 5 and Figure 6, A–C), indicating that TRAPα is crucial for ensuring proper PI folding and maintaining ER homeostasis when β cells are under stress. Moreover, the changes in islet cell composition, including increased proportions of GCG-positive cells, somatostatin-positive cells, and GCG double-positive cells (i.e., INS^+^/GCG^+^) (Figure 6, D–J), as well as upregulation of ALDH1A3 (Figure 6, K and L), further indicate that TRAPα deficiency may drive β cells toward dedifferentiation as they attempt to cope with stress. Taken together, these findings highlight the importance of TRAPα in preserving β cell function and identity, particularly under conditions of metabolic stress commonly seen in T2D. As such, underlying mechanisms regulating TRAPα expression in β cells and the potential link between TRAPα deficiency and compensation failure warrant further investigation. Additionally, it will be important to explore whether enhanced TRAPα expression or activity may rescue the observed phenotypes. This investigation could provide valuable insights into the specific role of TRAPα in compensatory processes within β cells and offer potential avenues for therapeutic intervention of T2D.

The traditional view of β cells as a homogenous population has evolved, with growing evidence suggesting significant heterogeneity in terms of gene expression, functional properties, and responses to physiological cues (41–43). Heterogeneity in β cell populations is considered a key aspect of the pathology of T2D, and variability in β cell gene expression and function among individuals is thought to contribute to differences in disease progression and treatment responses (44–47). Some β cells may exhibit impaired INS secretion or increased vulnerability to glucolipotoxicity — factors that are associated with the development and progression of T2D (48). In our study, we observed a significant reduction in detectable TRAPα protein within a subset of islet β cells in individuals with T2D. This decline in TRAPα was correlated with diminished INS content in those cells, accompanied by an increase in ER-localized PI. This correlation underscores a potential association between reduced TRAPα expression and impairment of INS production, which can certainly contribute to the pathophysiology of diabetes. Consistent with our previous report (18), identification of a distinct subpopulation of β cells with detectable PI but diminished INS introduces a layer of complexity to our comprehension of β cell maturation and function in the context of T2D. This subset of cells, characterized by inadequate INS content, aligns with existing literature emphasizing the heterogeneity of β cell populations in T2D. Further characterization of this β cell subpopulation is warranted to unravel the precise molecular mechanisms governing its contribution to overall islet dysfunction in the pathogenesis of T2D.

In conclusion, the present work advances our understanding by implicating an underappreciated mechanistic role for TRAPα in progressive INS deficiency in the pathogenesis of β cell dysfunction in T2D. The questions raised regarding failure of β cell compensation, β cell heterogeneity, and the impact of TRAPα deficiency under dietary stress provide a roadmap for future investigation. These findings not only contribute to the growing body of knowledge in the field, but they also hold the potential to guide the development of targeted therapeutic strategies for individuals with T2D.

## Methods

### Sex as a biological variable.

In this study, male and female mice were used, and the results were consistent between sexes. For human clinical samples, only male samples were used; however, the findings are expected to be consistent in both sexes.

### Mice.

TRAPα-floxed mice were generated by homologous recombination, with 2 *loxP* sites inserted flanking exon 2 of the TRAPα gene. The *Ins2*-IRES-Cre (JAX strain 037540) has been described (49). To generate TRAPα-βKO mice, TRAPα^fl/fl^ mice were crossbred with TRAPα^fl/fl^ *Ins2*-IRES-Cre^+/–^ mice. The genotype of control mice was TRAPα^fl/fl^, and the genotype of TRAPα-βKO mice was TRAPα^fl/fl^ *Ins2*-IRES-Cre^+/–^. The primers used for genotyping are listed in Supplemental Table 1. All mice were housed in a controlled environment with a 12-hour light/12-hour dark cycle, and temperature (22–25°C) and humidity (55% ± 5%) were maintained. All mice used in this study were on a C57BL/6J background. Body weight and blood glucose levels were monitored weekly at the same time of day to ensure consistency. For both in vivo and ex vivo experiments, all mice used in the same experiments were littermates.

### IPGTTs.

IPGTTs were performed by orally giving glucose 2 g/kg body weight to mice after 4 hours of fasting. Glucose measurements higher than 33.3 mmol/L were recorded as 33.3 mmol/L. Serum INS levels were determined with INS ELISA kits (EZassay) following the manufacturer’s protocol. All IPGTT experiments were performed in age- and sex-matched cohorts.

### Islet isolation and INS secretion assay.

Islet isolation was performed as previous described (20). For GSIS experiments, after overnight recovery, islets were incubated for 1 hour in HEPES balanced Krebs-Ringer bicarbonate buffer (KRBH) (135 mmol/L NaCl, 3.6 mmol/L KCl, 0.5 mmol/L MgSO_4_·7 H_2_O, 0.5 mmol/L NaH_2_PO_4_, 2 mmol/L NaHCO_3_, 10 mmol/L HEPES, 1.5 mmol/L CaCl_2_, 0.1% BSA) without glucose. Thereafter, the islets were incubated in KRBH containing a low concentration of glucose (2.8 mmol/L) for 2 hours, followed by a stimulatory glucose (16.7 mmol/L) for additional 2 hours. At the end of the stimulation, supernatants were collected, and the islets were homogenized in acid alcohol. Secreted INS and INS in the islets were measured using ELISA kits (EZassay). The secretion efficiency was calculated with secreted INS in the media normalized to islet INS content.

### Immunofluorescence and immunohistochemical staining.

Paraffin-embedded human and mouse pancreases were cut into 5 μm sections. Immunohistochemical staining was performed using appropriate antibodies as previously described (20). The antibodies used in this study were as follows: anti-TRAPα (catalog NBP1-86912; Novus Biologicals), anti-PI/CCI-17 (catalog NB100-73013; Novus Biologicals), anti-INS (homemade), anti-GCG (catalog G2654, MilliporeSigma), and anti-somatostatin (catalog ab30788; Abcam). Fluorescent images were visualized using Axio Imager M2 (Carl Zeiss).

### RNA extraction, cDNA synthesis, and qRT-PCR.

Total RNA of islets was extracted using TRIzol reagent (Invitrogen), and cDNA was synthesized using a PrimeScript RT reagent Kit with gDNA Eraser (Takara Bio; catalog RR047A). Real-time PCR was conducted with the TB Green Premix Ex Taq (Tli RNaseH Plus) (Takara Bio; catalog RR420A). *GAPDH* was used as internal control. Primers were designed and chemically synthesized by TSINGKE Biological Technology Co. The sequence information of the primers used in this study are listed in Supplemental Table 2.

### Protein extraction and Western blot analysis.

Isolated islets were lysed in RIPA buffer. Equal amounts of protein samples were loaded on four 12% NuPage gradient gels (Thermo Fisher Scientific), transferred to nitrocellulose membrane (BioTrace NT nitrocellulose membrane, catalog 66485; PALL), and blotted with appropriated first antibodies followed by secondary antibodies conjugated with HRP. The antibodies used in this study were as follows: anti-PI/CCI-17 (catalog NB100-73013; Novus Biologicals), anti-INS (homemade), anti–β-tubulin (catalog KM9003; Sungene), anti-GAPDH (catalog AC033; Abclonal), anti-CPE (catalog GTX33060; GeneTex), anti-TRAPα (homemade), anti-TRAPβ (homemade), anti-TRAPγ (homemade), anti-TRAPδ (homemade), anti-PDI (catalog ab2792; Abcam), anti-BiP (catalog sc-376768; Santa Cruz Biotechnology), anti–p-eIF2α (catalog 9721S; Cell Signaling Technology), anti–t-eIF2α (catalog 9722S; Cell Signaling Technology).

### Massive parallel RNA-Seq and analysis.

The islet transcriptome analysis was conducted by OE Biotech Co. Total RNA was extracted using the mirVana miRNA Isolation Kit (Ambion). RNA integrity was evaluated using the 2100 Bioanalyzer (Agilent Technologies). The samples with an RNA integrity number ≥7 were subjected to the subsequent analysis. The libraries were constructed using TruSeq Stranded mRNA LTSample Prep Kit (Illumina) according to the manufacturer’s instructions. Then these libraries were sequenced on the Illumina sequencing platform (HiSeq 2500 or Illumina HiSeq X Ten) and 125 bp/150 bp paired-end reads were generated. Raw data (raw reads) were processed using Trimmomatic. The reads containing poly-N and the low-quality reads were removed to obtain the clean reads. Then the clean reads were mapped to reference genome using hisat2. The differential expressed genes were identified using the absolute value of log_2_ (ratio) ≥1 as the threshold. Gene Ontology analysis and pathway enrichment studies were performed by WebGestalt, and pathway visualization was conducted using GraphPad Prism 9.

### Ablation of Ins1 and Ins2 genes in INS1 cells.

CRISPR/Cas9–mediated ablation of *Ins1* and *Ins2* genes of INS1 cells (INS1 MUT) was achieved with CRISPR/Cas9 RNP (provided by Haixing Bioscience) containing expression cassettes for hSpCas9 and chimeric guide RNA. To target exon 1 of *Ins1* and exons 1~2 of the *Ins2* gene, 4 guide RNA sequences — CCACCAGGTGAGGACCACAAAGG, CGGGTCCTCCACTTCACGACGGG, AGGGATTTGAGGGACGCTGTGGG, and TCATTGCAGAGGGGTGGACAGGG — were selected through the Zhang lab website (https://www.zlab.bio/resources). Plasmids containing the guide RNA sequences were electrotransfected into INS1 WT cells (INS1 WT) using the Neon transfection system according to the manufacturer’s instructions (Thermo Fisher Scientific). After 2 days, transfected cells were transferred into 96-well plates. Having grown a single clone, the DNA was isolated and used to determine deletions of targeted *Ins1* and *Ins2* exons using 2 pairs of primers. The first pair of the primer sequences was as follows: forward: 5′-ACCCAGTAACTCCCAACCCT-3′, reverse: 5′-AGTGGCATTTACCCTTTGACTCT-3′; the second pair of primer sequences was as follows: forward: 5′-GCTCTGAAGCAAGCACCTCT-3′, reverse: 5′-TGGGAGGAGGGACTGAGAAG-3′. The expression of PI and INS in the clones with mutations were further confirmed by Western blotting using anti-INS. The clones with confirmed INS deletions in both gene and protein levels were selected for downstream studies. Cell lines generated in this study include control cells (INS1 WT) and *Ins1/Ins2* KO cells (INS1 MUT). All clones were maintained under the same conditions as INS1 parental cells.

### Statistics.

All experiments described in this report were performed independently at least 3 times. All data are presented as mean ± SEM. GraphPad Prism 9 software was used for all statistical analyses. We analyzed data using an unpaired Student’s *t* test or 2-way ANOVA, and we used the traditional threshold of *P* < 0.05 to declare statistical significance.

### Study approval.

All animal experiments followed the protocols approved by the Internal Animal Welfare Committee at Tianjin Medical University. Human pancreas tissues were obtained between January 2016 and January 2023 from organ donors with or without T2D (Supplemental Table 3) after written informed consent was obtained. Studies using human pancreases were approved by Tianjin First Central Hospital clinical research ethics Committee (no. 2016N082KY).

### Data availability.

Raw values for data presented in figures and tables are provided in the Supporting Data Values file. Additional data are available upon request. The raw sequencing data supporting the findings of this study have been deposited in the NCBI Sequence Read Archive (SRA) under the BioProject accession number PRJNA1261638. These data are publicly available and can be accessed through the NCBI SRA database.

## Author contributions

X Li, JH, and YH designed and conducted experiments and analyzed data; HZ, NX, YL, X Liu, YY, XZ, XX, WF, YF, and ZZ conducted experiments; SW and WJZ provided reagents; PA contributed to discussions about the project and edited manuscript; and ML initiated and oversaw the project, designed the experiments, and wrote the manuscript.

## Supplementary Material

Supplemental data

Unedited blot and gel images

Supporting data values

## Version 1 05/20/2025

In-Press Preview

## Version 2 07/15/2025

Electronic publication
