# Expression of Concern for Trapα deficiency impairs the early events of insulin biosynthesis and glucose homeostasis

**DOI:** 10.1172/JCI213416

**Published:** 2026-10-01

**Authors:** Xin Li, Jingxin Hu, Yumeng Huang, Hai Zhang, Ning Xu, Yang Liu, Xuan Liu, Yuanyuan Ye, Xinxin Zhang, Xiaoxi Xu, Yuxin Fan, Ziyue Zhang, Weiping J. Zhang, Shusen Wang, Wenli Feng, Peter Arvan, Ming Liu

Original citation: *J Clin Invest*. 2025;135(14):e179845. https://doi.org/10.1172/JCI179845

Citation for this expression of concern: *J Clin Invest*. 2026;136(19):e213416. https://doi.org/10.1172/JCI213416

The authors recently alerted the Editors to errors in the source data and/or figures for 2B, 2F, 2H, 3A, 3J, S4D, and S7A that were identified in a post-publication audit of the data. Although the authors have indicated that the errors were due to inadvertent mistakes, the Editors are issuing this Expression of Concern to alert readers to the issues. We have requested institutional oversight into this matter and will alert readers to the outcome when the institutional evaluation is complete.

## Version 1 10/01/2026

Electronic publication

