# Supplementary material for: Trapα deficiency impairs the early events of insulin biosynthesis and glucose homeostasis
Source: J Clin Invest. 2025 May 20;135(14):e179845. doi: 10.1172/JCI179845 (PMC12259251; doi:10.1172/JCI179845)
Supplement: Supplemental data [file jci-135-179845-s009.pdf]

Fig.S1

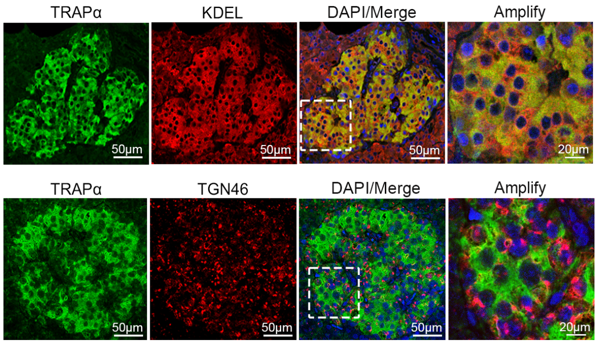

Fig. S1. Sub-cellular localization of TRAPα in human pancreas. Confocal microscopy images of human pancreatic sections co-immunostained with anti-TRAPα (green) and anti-KDEL (an ER marker, red, *upper panel*), or with anti-TGN46 (a Trans-Golgi Network marker, red, *bottom panel*). TRAPα shows strong colocalization with KDEL.

Fig.S2

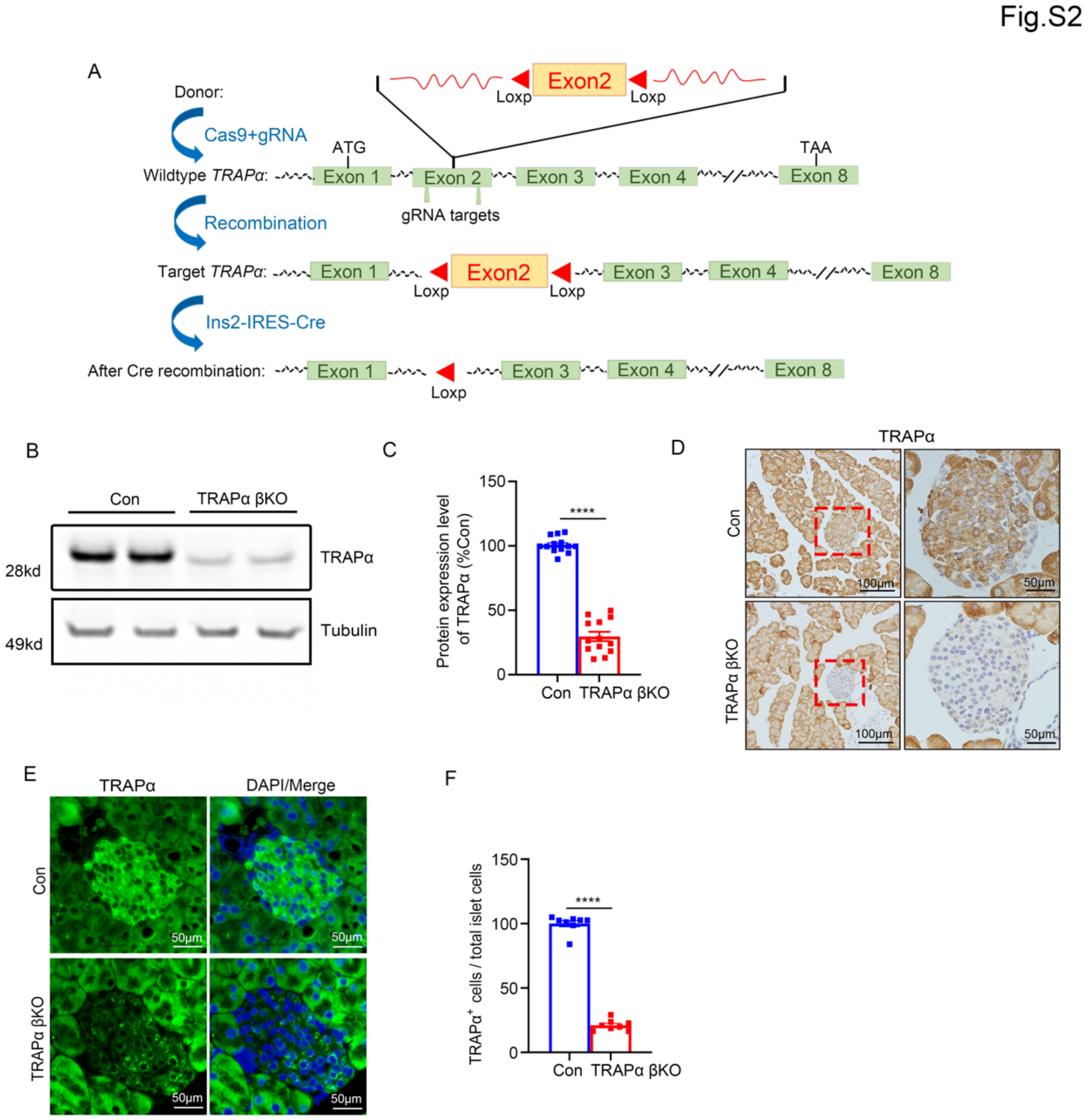

Fig. S2. Generation and confirmation of TRAPα-βKO mouse line. A. Schematic diagram showing deletion of the exon 2 of TRAPα gene by *Ins2*-IRES-Cre. **B.** Representative Western blot showing TRAPα expression in islets isolated from 8-12-week-old Con and TRAPα-βKO male mice. **C.** quantification of TRAPα protein levels based on Western blot analysis in B (n = 13). **D.** Immunohistochemistry staining was performed to detect the expression of TRAPα in pancreases of 8-12-wk-old Con or TRAPα-βKO male mice. **E.**Pancreatic sections of 8-12-wk-old Con and TRAPα-βKO male mice were immunoblotted with anti-TRAPα (Green) as

indicated. **F.** TRAPα positive (TRAPα$^+$) cells shown in (E) were counted in Con and TRAPα-βKO islets. Percentages of TRAPα positive cells in Con islets were calculated and the percentage of TRAPα positive cells was set as 100%. TRAPα-βKO resulted in 79% reduction of TRAPα$^+$ cells in islets (n=8). Values are shown as mean ± SEM. Statistical significance is indicated as ****$P < 0.0001$.

Fig.S3

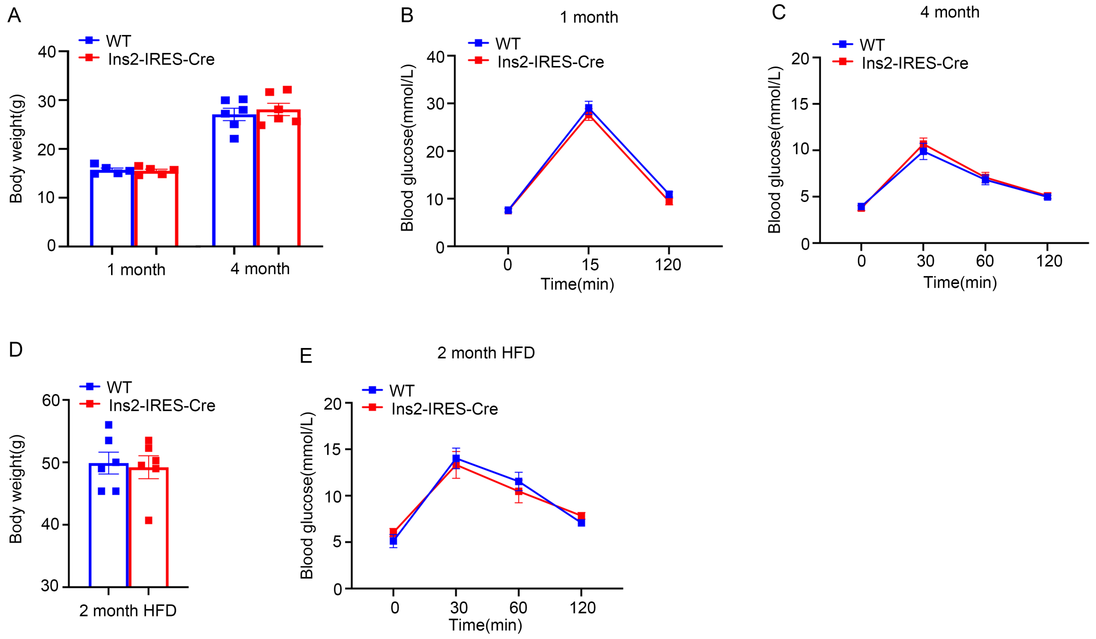

Fig. S3. *Ins2*-IRES-Cre mice exihit same body wight and glucose tolerance as wild-type control mice fed with chow diet or HFD. A. Body weight of C57BL/6J (WT) and C57BL/6J-*Ins2*-IRES-Cre male mice on chow diet at 1 month and 4 months of age (n=5-6). **B.** Glucose tolerance as measured by IPGTT in 1-month-old WT and *Ins2*-IRES-Cre male mice on chow diet (n=5). **C.** Glucose tolerance as measured by IPGTT in 4-month-old WT and *Ins2*-IRES-Cre male mice on chow diet (n=6). **D.** Body weight of WT and *Ins2*-IRES-Cre male mice fed with chow diet for 2 months followed by additional 2 months of HFD feeding (n=6). **E.** Glucose tolerance as measured by IPGTT in WT and *Ins2*-IRES-Cre male mice fed with chow diet for 2 months followed by additional 2 months of HFD feeding (n=6).

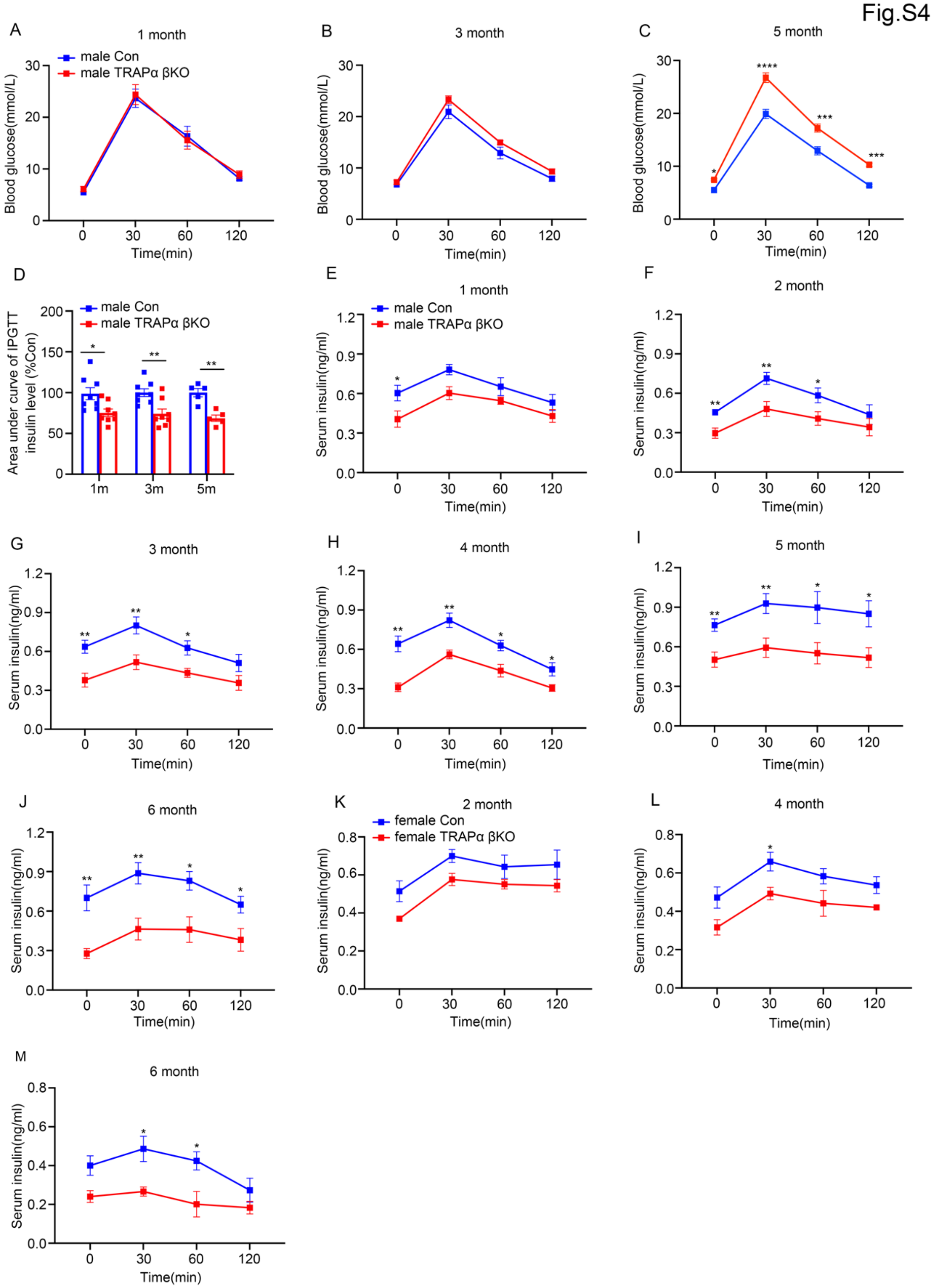
Fig.S4
A
1 month
male Con
male TRAPα βKO
Blood glucose(mmol/L)
Time(min)
B
3 month
C
5 month
D
Area under curve of IPGTT insulin level (%Con)
1m
3m
5m
E
1 month
Serum insulin(ng/ml)
F
2 month
G
3 month
H
4 month
I
5 month
J
6 month
K
2 month
female Con
female TRAPα βKO
L
4 month
M
6 month

Fig. S4. TRAPα-βKO decreases circulating insulin and impairs glucose intolerance in male and female mice. A-C. IPGTT were performed in Con and TRAPα-βKO male mice at 1-, 3-, and 5-month-old (n=6-10). **D.** The AUC for insulin levels of the same group mice as (A-C). **E-J.** Raw serum insulin levels of monthly IPGTT (1-6 months) in Con and TRAPα-βKO male mice (n=6-7). **K-M.** Raw serum insulin levels of during IPGTT at 2-, 4-, and 6- month-old Con and TRAPα-βKO female mice (n=3-4). Values were shown as mean ± SEM. *P < 0.05, **P < 0.01, ***P < 0.001 and ****P < 0.0001.

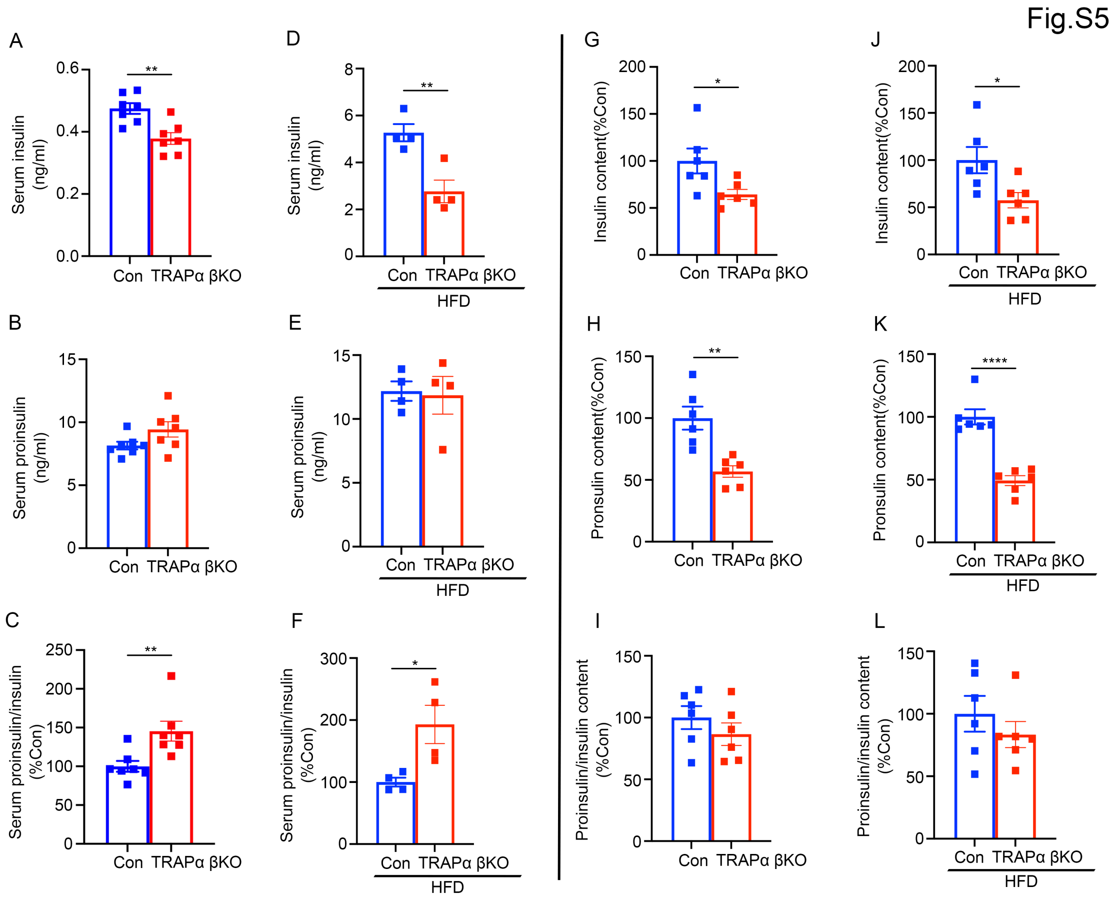

Fig. S5. TRAPα-βKO causes decreased serum and islet insulin levels and increased ratio of circulating proinsulin and insulin. A–C. Serum insulin (A), proinsulin (B), and proinsulin/insulin ratio (C) in Con and TRAPα-βKO male mice fed chow diet for 4 months. Panels **D–F**. Serum insulin (D), proinsulin (E), and proinsulin/insulin ratio (F) in Con and TRAPα-βKO male mice fed with 2-month chow diet followed by additional 2-month HFD feeding. Panels **G–I**. Islet insulin (G), proinsulin (H), and proinsulin/insulin ratio (I) in Con and TRAPα-βKO male mice fed with chow diet for 4 months. Panels **J–L**. Islet insulin (J), proinsulin (K), and proinsulin/insulin ratio (L) in Con and TRAPα-βKO male mice fed with 2-month chow diet followed by additional 2-month HFD feeding. Values are shown as mean ± SEM. n=4-7. Statistical significance is indicated as $^*P < 0.05$, $^{**}P < 0.01$ and $^{****}P < 0.0001$.

Fig.S6

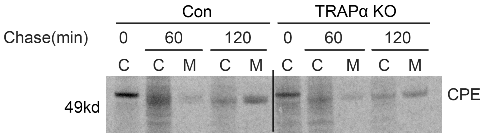

Fig. S6. TRAPα deficiency does not affect secretion of newly synthesized CPE. INS832/13 control (Con) and TRAPα knockout cells (TRAPα KO) were pulse-labeled with ^{35}S-Met/Cys for 20 minutes followed by chase for indicated times. Newly synthesized CPE from cell lysates “C” and media “M” were immunoprecipitated and analyzed by SDS-PAGE under reducing conditions.

Fig.S7

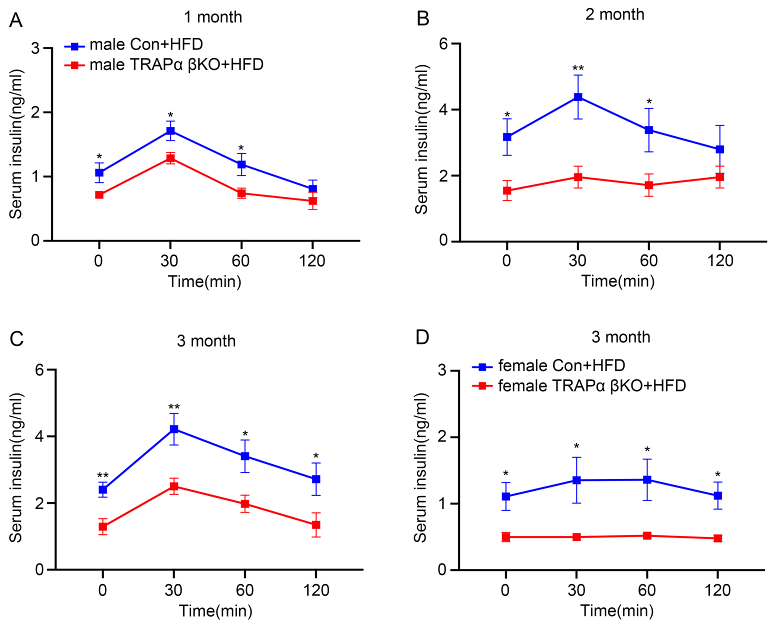

Fig. S7. TRAPα-βKO leads to decreased circulating insulin during IPGTT after 3 months of HFD. A-C. The raw non-normalized serum insulin levels of male mice shown in Fig. 5I. **D**. The raw non-normalized serum insulin levels during IPGTT in Con and TRAPα-βKO female mice fed with chow diet for 2 months followed by 3 month HFD feeding (n=5). The glucose levels during IPGTT were shown in Fig. 5H. Values are expressed as mean ± SEM. *P < 0.05 and **P < 0.01.

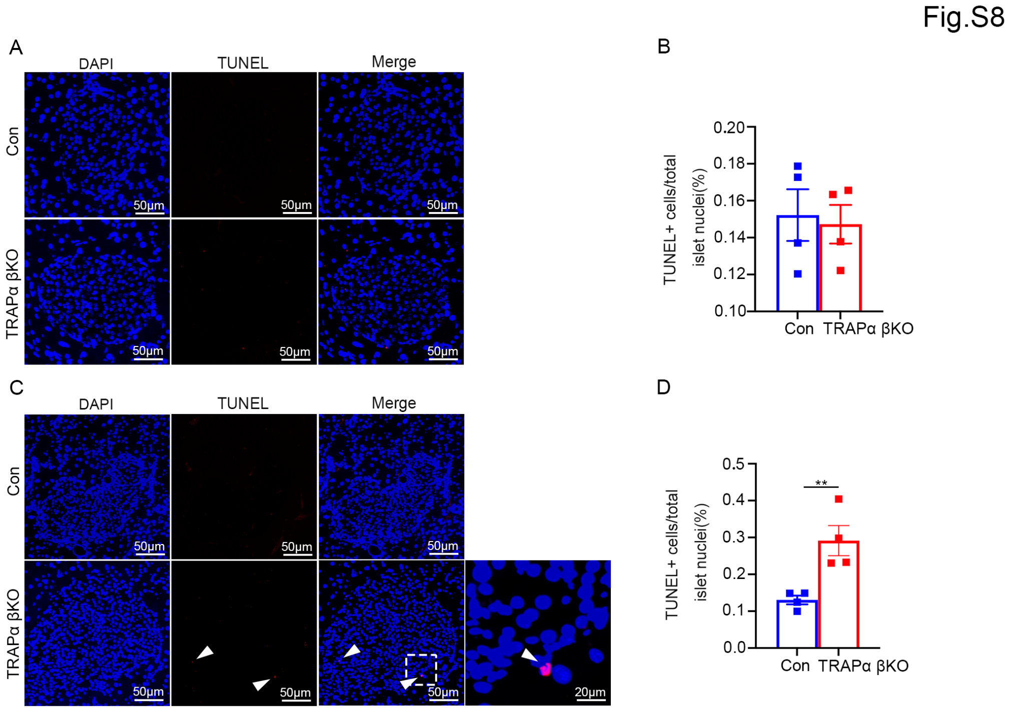

Fig. S8. TRAPα deficiency leads to increased β cell apoptosis in mice fed with HFD. A. TUNEL staining of pancreatic islets from Con and TRAPα-βKO male mice fed with chow diet. Nuclei were stained with DAPI (blue), and apoptotic cells were indicated by TUNEL-positive signals (red). **B**. Quantification of TUNEL-positive cells in islets shown in (A) n=4. **C**. TUNEL staining of pancreatic islets from Con and TRAPα-βKO male mice fed with chow diet for 2 months followed by additional 3 month of HFD feeding. Nuclei were stained with DAPI (blue), and apoptotic cells were marked by TUNEL-positive signals (red). Arrowheads indicated TUNEL-positive cells. **D**. Quantification of TUNEL-positive cells in islets shown in (C). n=4. Values are shown as mean ± SEM. Statistical significance was indicated as $^{**}P < 0.01$.

Supplemental Table 1. Primers for genotyping (5'-3')

Ins2-IRES-Cre forward	CCCGCCGTGAAGTGGAGGAC
Ins2-IRES-Cre reverse	CAGCCCGGACCGACGATGAA
TRAPα forward	GATAGTGTTTTCTCTTGGGCTTTGCAC
TRAPα reverse	TTATTACCACTTCTCGGGGAGCGA

Supplemental Table 2. Primers for qRT-PCR (5'-3')

Gene	Forward	Reverse
GAPDH	AAGAGGGATGCTGCCCTTAC	TACGGCCAAATCCGTTCACA
Canx	CTGGCAGTCAAGATGAGGAAG	GCCCTCTCCTAGGACCACAT
Calr	CCGGGACAATCTTTGACAAT	CCTCATCCTGCTTGTCCTTC
Sar1b	TTCCTTGGATTGGATAATGCCG	GCCAGCAATAGTAAGCTCTTCTG
Bip	TGTGTGTGAGACCAGAACCG	TAGGTGGTCCCCAAGTCGAT
chop	CTTGAGCCTAACACGTCGAT	AGGTTCTGCTTTCAGGTGTG
Derlin	GCAACTCGGTTGTCACAGACCT	CTGAGGGTCATCTAGTAGCAGC
Hrd1	AGCTACTTCAGTGAACCCCACT	CTCCTCTACAATGCCCACTGAC
Sel1l	TGGGTTTTCTCTCTCTCCTCTG	CCTTTGTTCCGGTTACTTCTTG
Ero1la	AGGCTGTTCTTCAGTGGACG	CATACTCAGCATCGGGGGAC
Ero1lb	ACAAAAAGGGGGCCAAGTCA	CTCTCCTGAGTATCCAGCAGTT
Eif2a	CACGGTGCTTCCCAGAGAATC	GTCCCTTGTTAGCGACATTGA
Pdia1	GGTGAGCGGACACTAGATGG	AGGTCGAGGTCGTCATTGTC
Pdia2	TCACTGACCAGCCACAACTT	CCGACCCTCATCAAACTTCTTG
Pdia3	GTGTGGACATTGCAAGAGGC	TTGGCAGTGCAATCCACCTTT
Pdia4	CACAGGCCTCTTGGATGTGA	TGCAGTGTCCACACCAAATCTT
Pdia5	ATTTCGAGAAGGGGCGGTTT	CCGTTCTGCCACCCTGATAA
Pdia6	TTGACAGTGAAGACGTTTGGATG	GCCAGAACCTGATTCATGGTG
Erp27	TCCTCTCGTTTGTCCTCGTG	GTAGTGCTGAGACCATCCGT
Erp29	TTCGACACCCAGTACCCCTAT	GCTTGTCGCCATAGTCTGA
Erp44	TCCTTGCTGCTCCTGGTAACTT	CTGGGCTATATCAGCAGTCAGC
Tmx1	TGGTTTGGTCCAAGTTCTGT	GCTGTGGCTAGTCCTGATGT
Tmx2	CGTGTGACTTTGACTGGAGAGA	TCAGGAACATGGATCTGCGG

Tmx3	TTACCGAGGACCACGGACTA	CCGAATTAGAGCCCCAGACA
Tmx4	CTGGCAAGATTTGGCACCTTC	ACACCAATGTGGCAATGACG
Ins1	CTTCTTCTACACACCCAAGTCC	CAGCTCCAGTTGTTCCACTT
Ins2	GAAGTGGAGGACCCACAAG	GTCTGAAGGTCACCTGCTC
TRAPα	TGGCTCAAGATCCTACAGAAGA	TCTGTGGGTTCATCTTCTTCTACTT
TRAPβ	TTGGCTCTGTTAGCCGTCAG	TTGAGAGGACGCAGGACAAC
TRAPγ	GAGGGAGGATGCTGTTTCCA	AGACAAGAGGGCGATGAGTC
TRAPδ	GCCTGGAACCACAGATCACC	GACCCTGTTCTTGCAGGTCA

Supplemental Table 3. The information of donors.

	Con	T2D
N number	5	5
Age	50.8±7.7	55.4±5.9
Sex	male	male
BMI	21.1±1.8	25.2±2.6
HbA1c(%)	5.1±0.6	7.9±2.4
