# Supplementary material for: Trapα deficiency impairs the early events of insulin biosynthesis and glucose homeostasis
Source: J Clin Invest. 2025 May 20;135(14):e179845. doi: 10.1172/JCI179845 (PMC12259251; doi:10.1172/JCI179845)

# Full unedited gel for Figure 1D

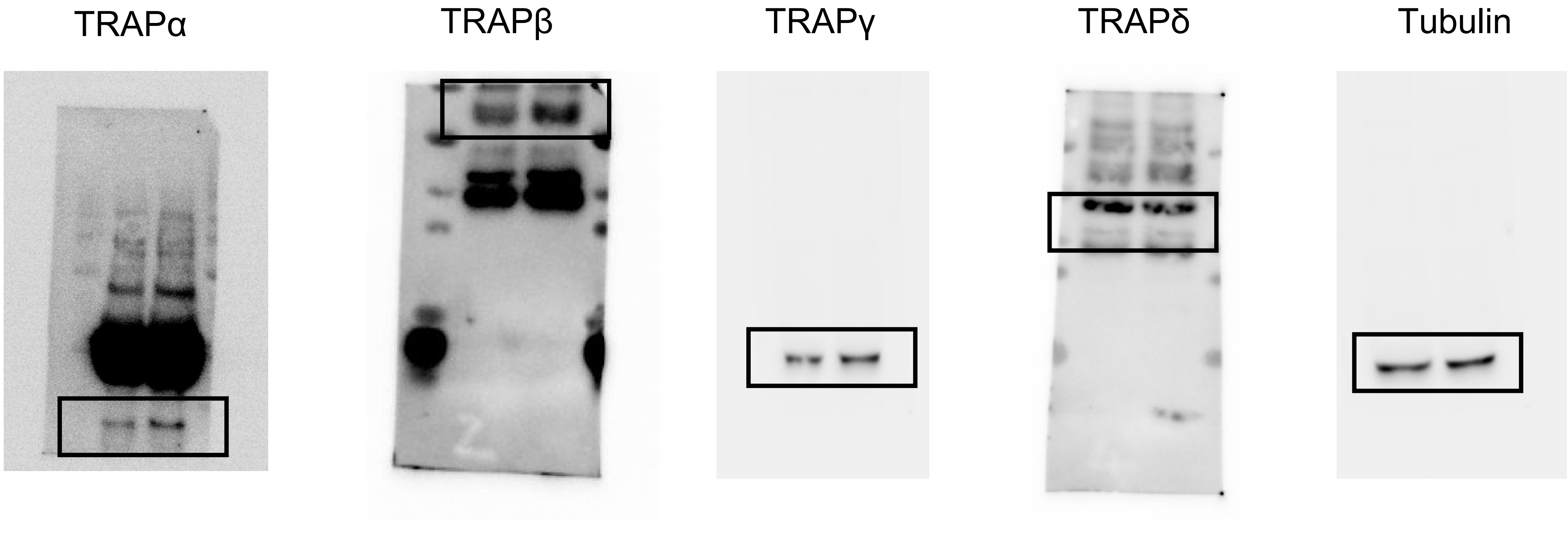

Full unedited gel for Figure 1G

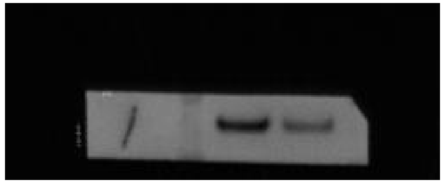

TRAPα

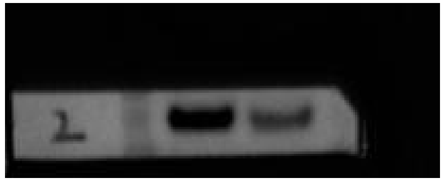

TRAPβ

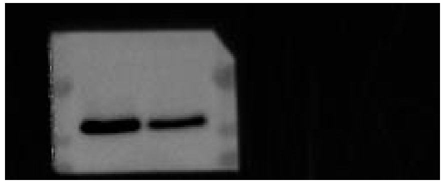

TRAPγ

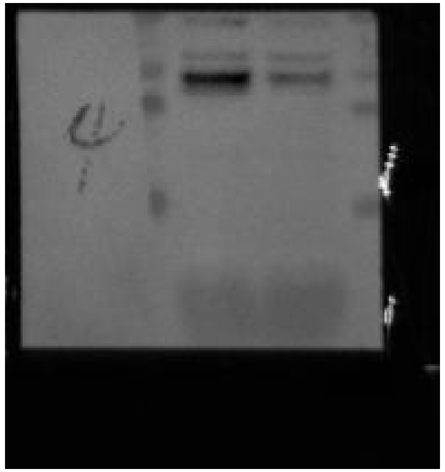

TRAPδ

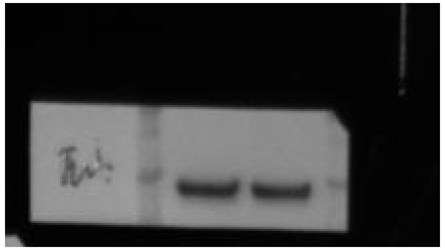

Tubulin

# Full unedited gel for Figure 3H

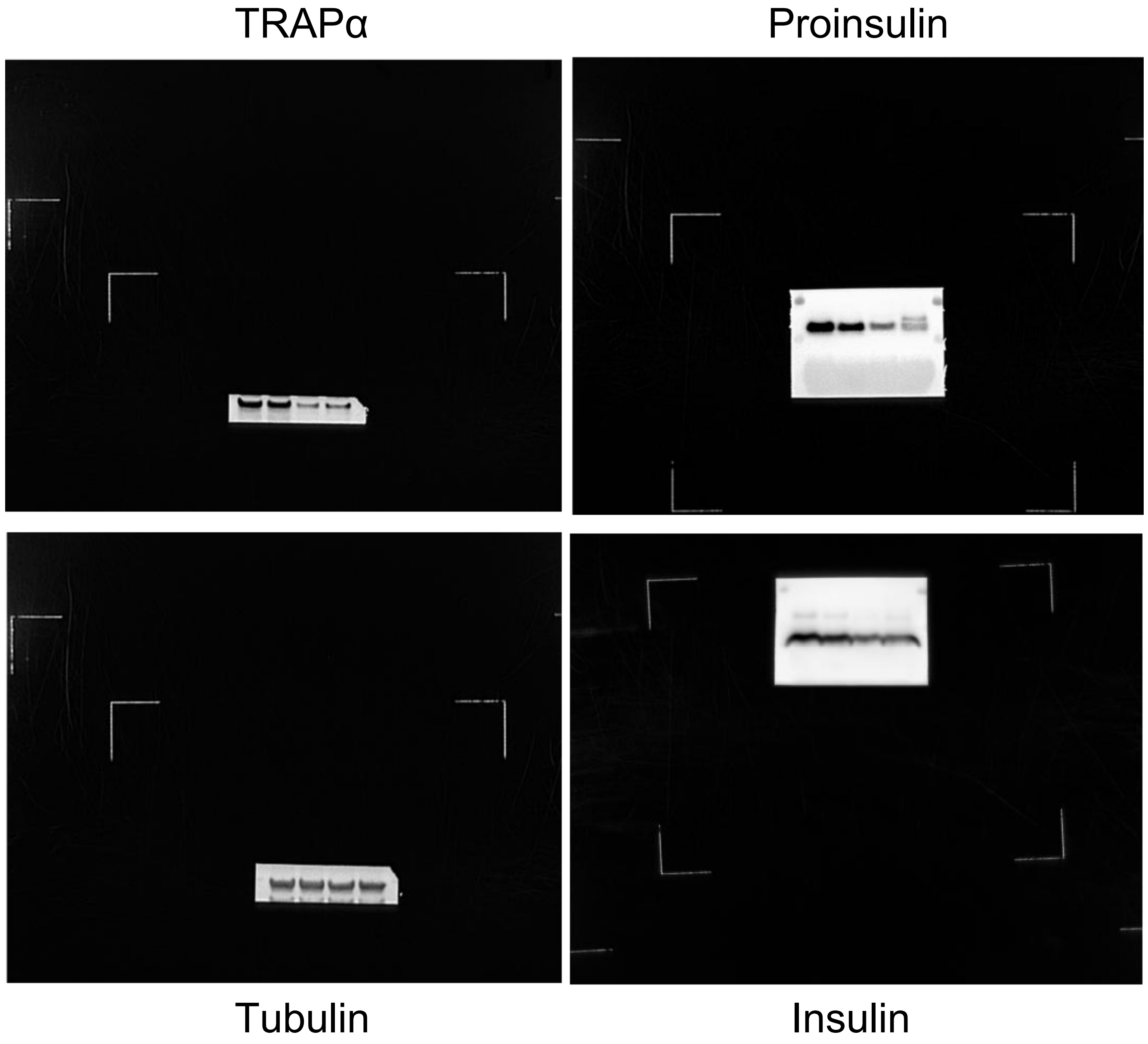

# Full unedited gel for Figure 4D

t-eIF2α

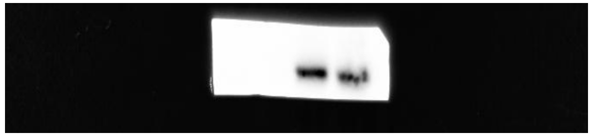

p-eIF2α

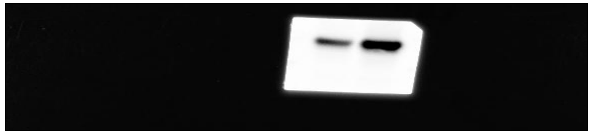

BiP

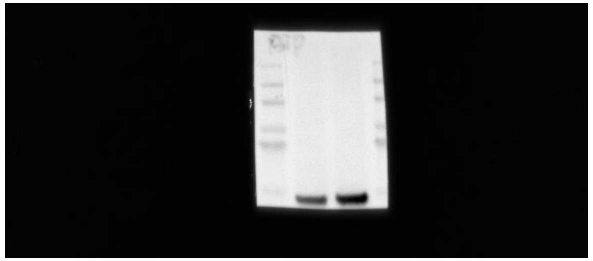

Tubulin

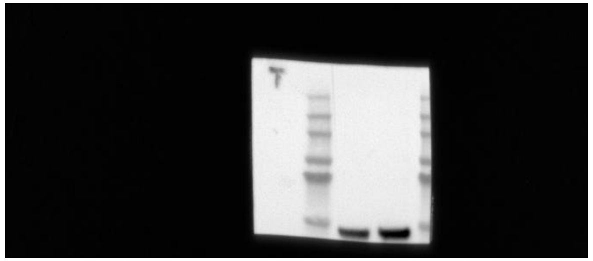

# Full unedited gel for Figure 4F

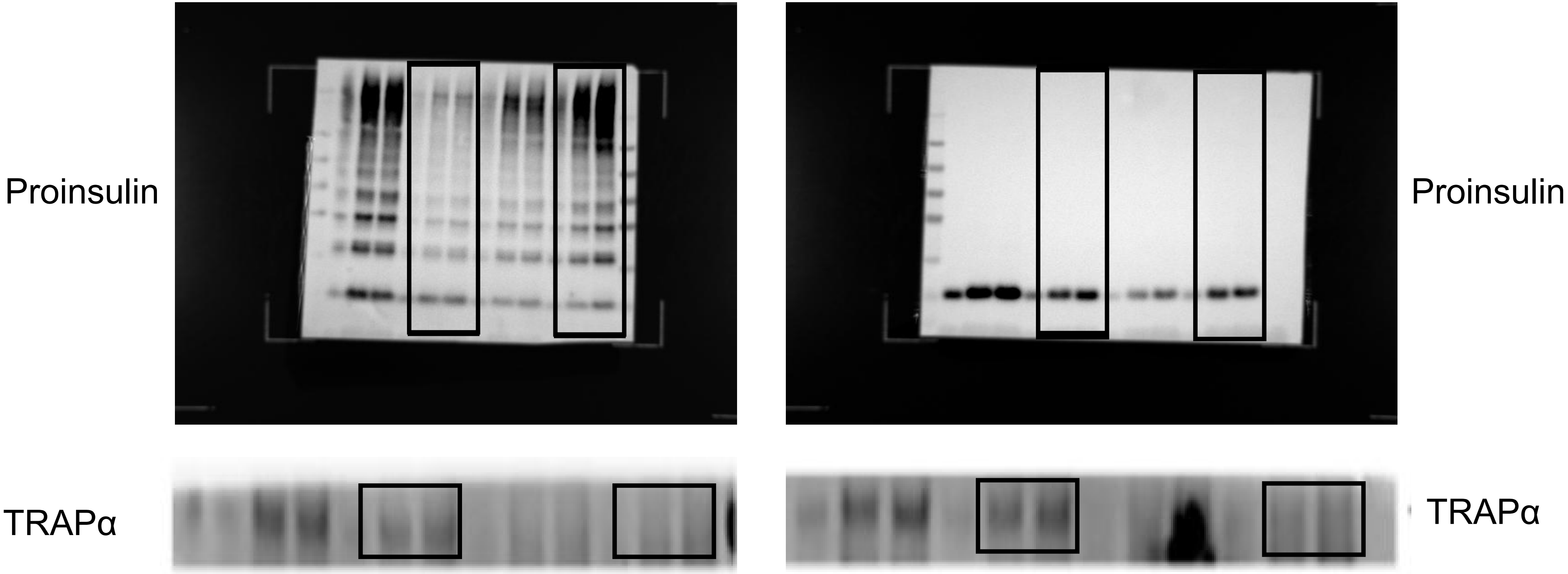

# Full unedited gel for Figure 4F

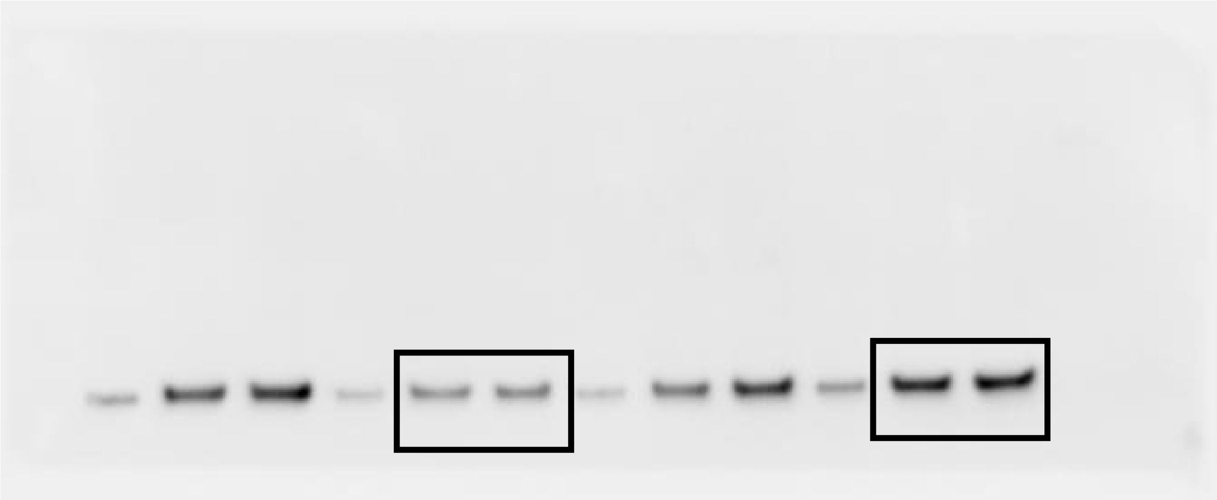

Tubulin

# Full unedited gel for Figure 4H

BiP

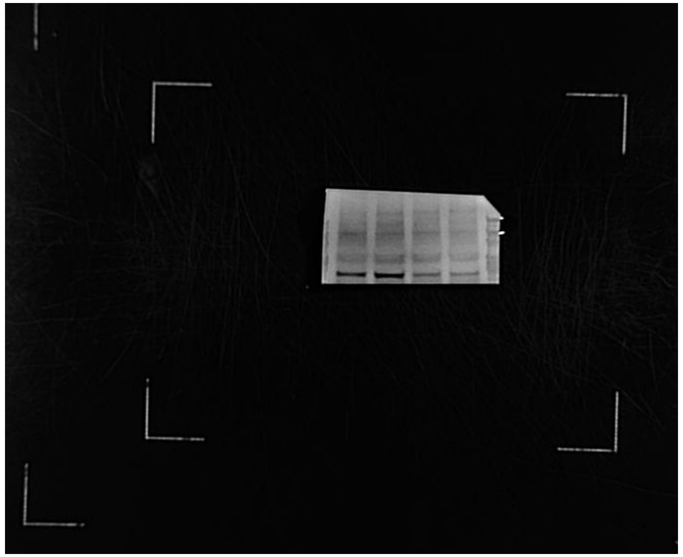

t-eIF2α

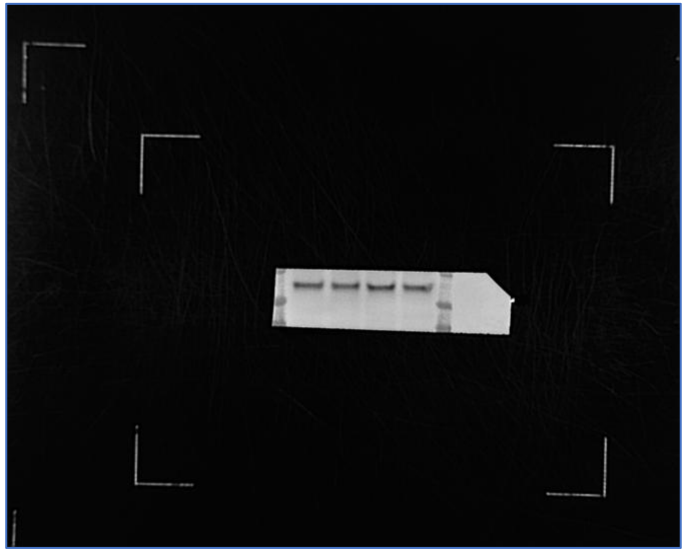

p-eIF2α

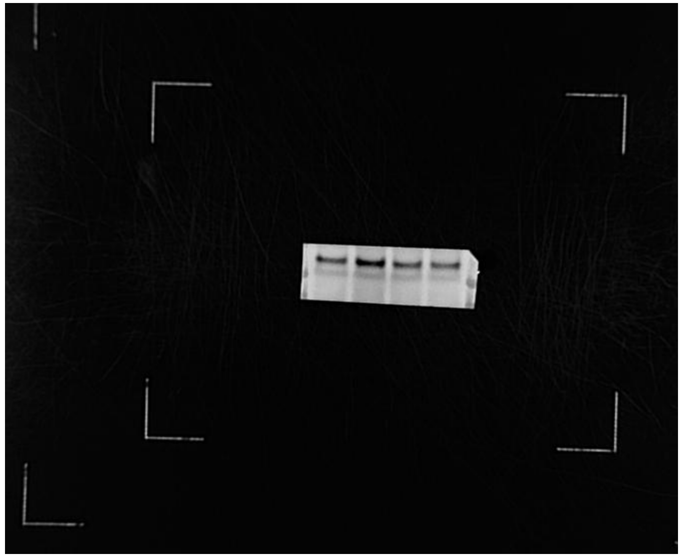

Proinsulin/Insulin

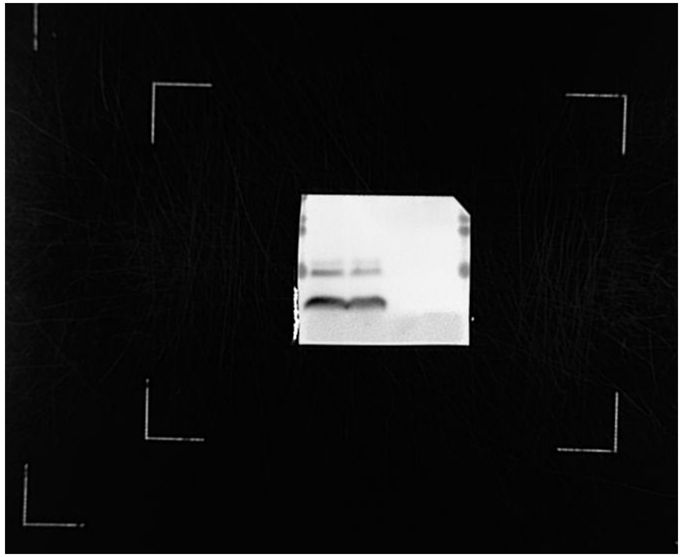

TRAPα

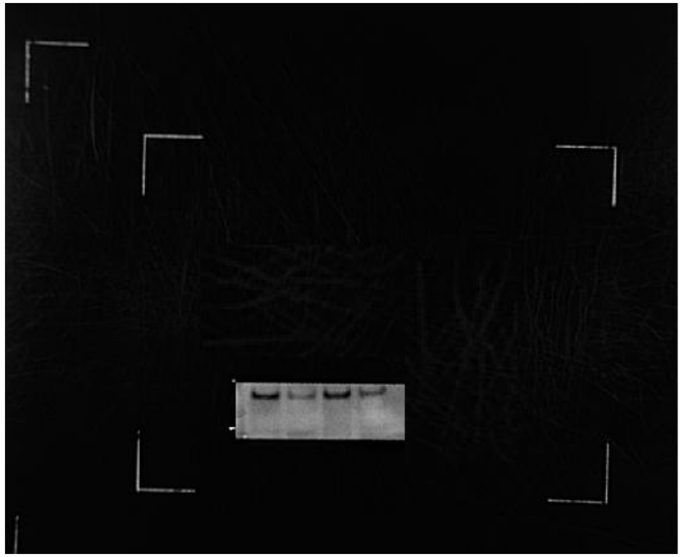

Tubulin

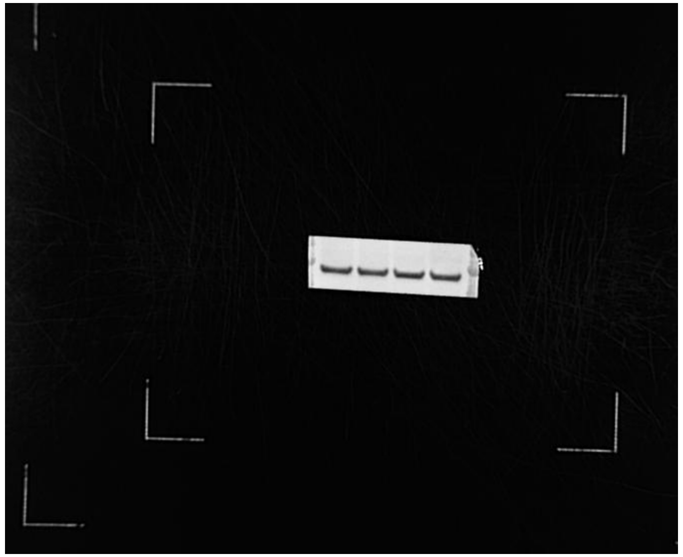

# Full unedited gel for Figure 4J

CPE

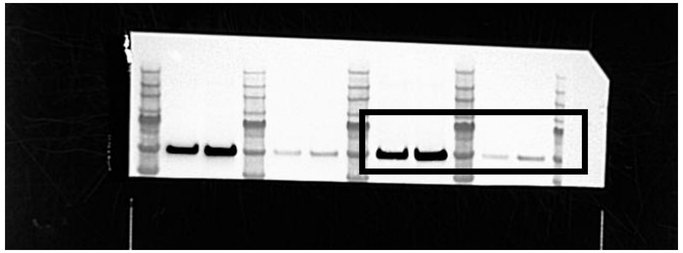

Proinsulin

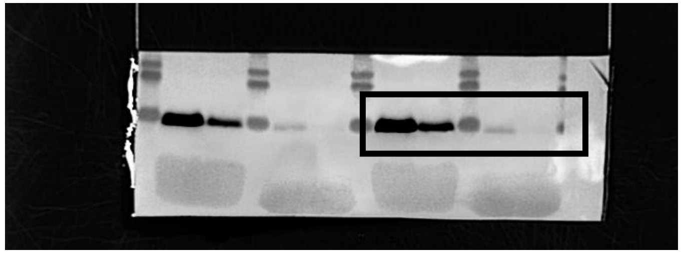

Tubulin

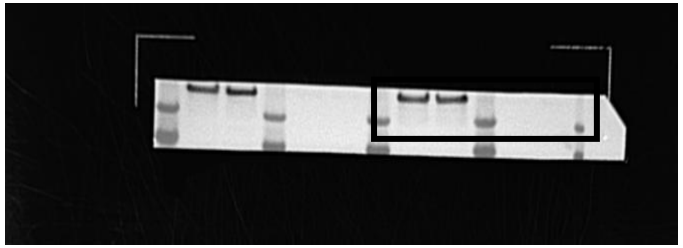

# Full unedited gel for Figure 5J

TRAPα

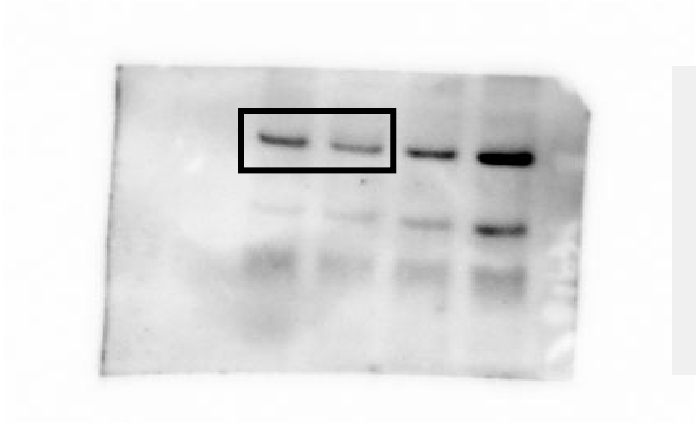

Proinsulin/Insulin

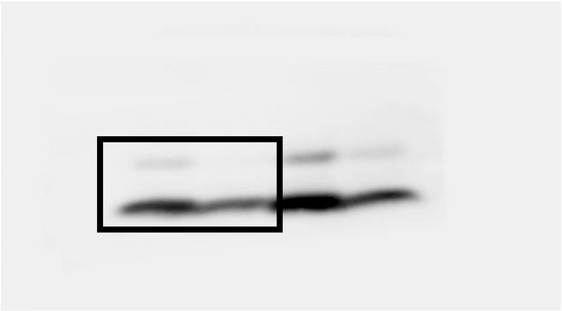

Tubulin

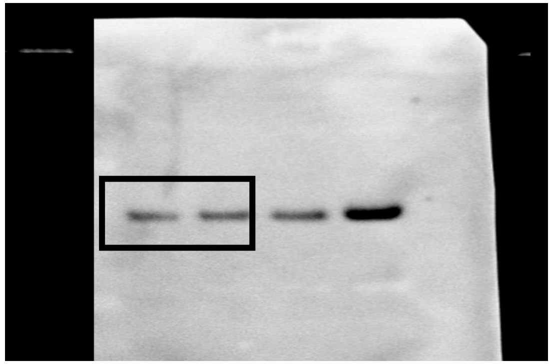

# Full unedited gel for Figure 6A

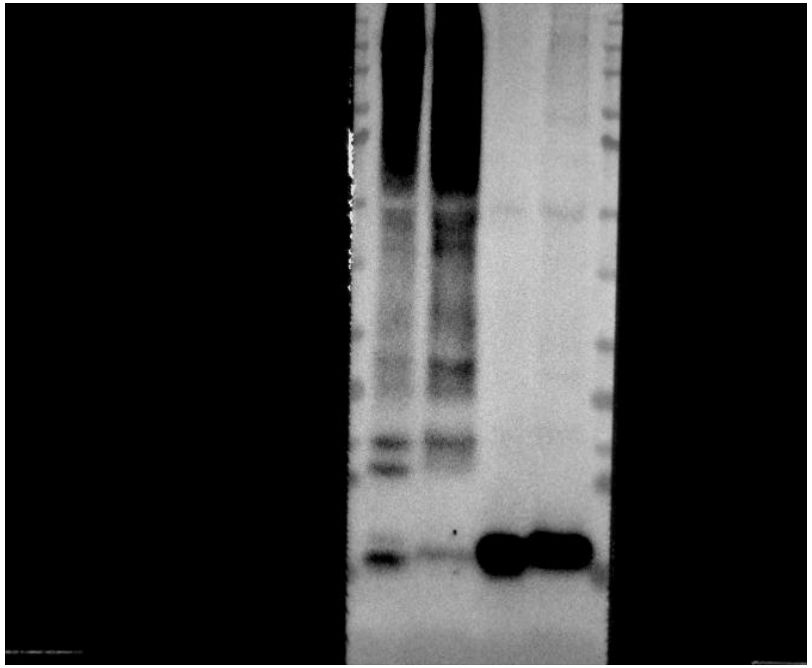

Proinsulin

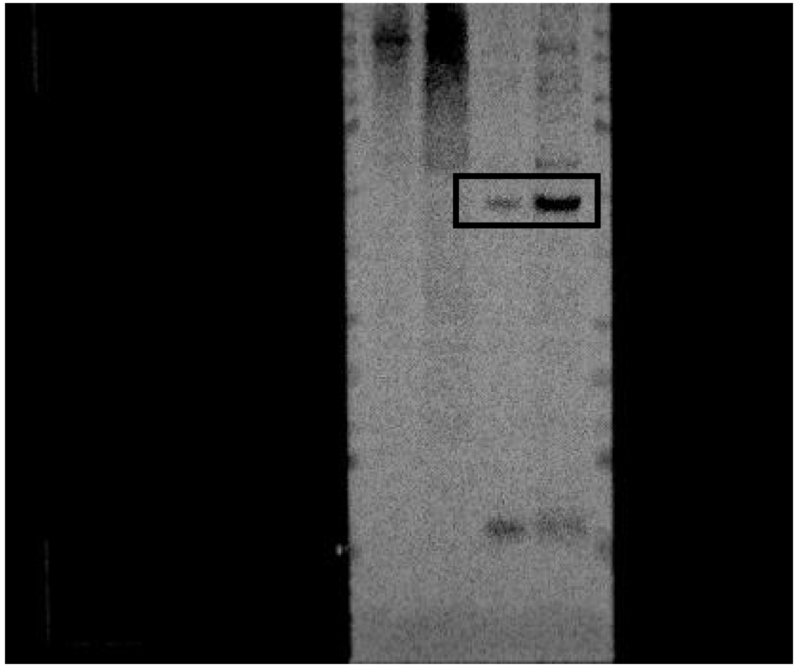

Tubulin

# Full unedited gel for Figure 7J

TRAPα

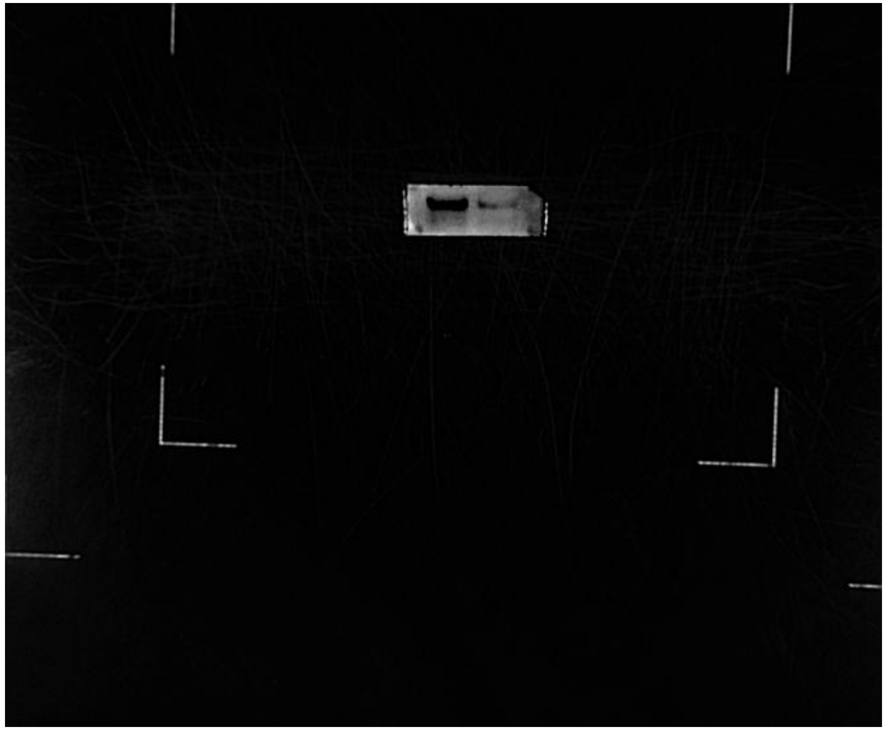

GAPDH

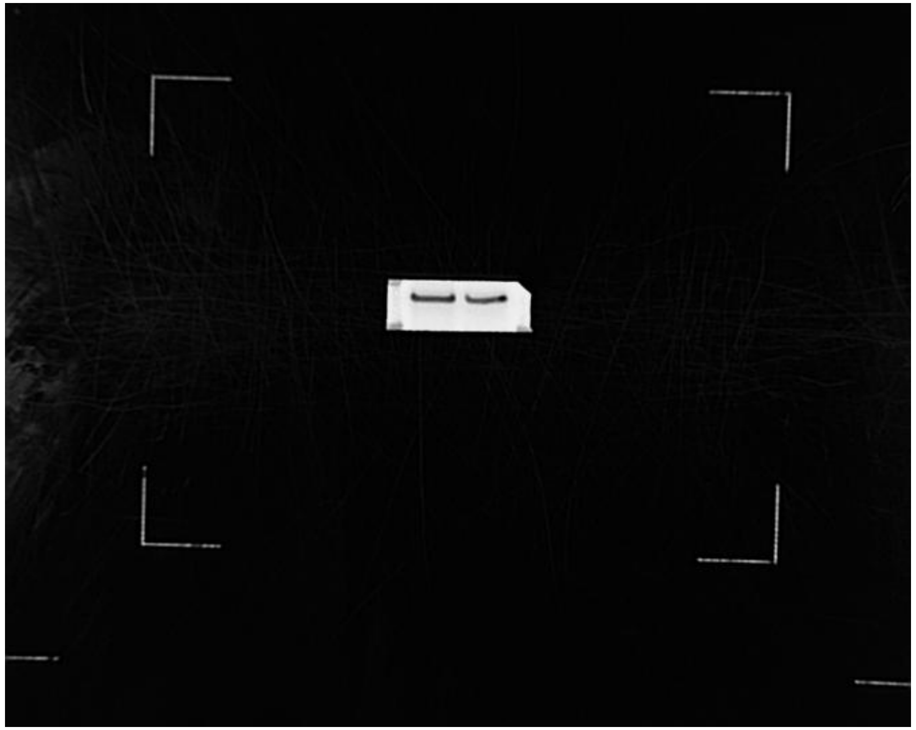

# Full unedited gel for Supplemental Figure S2B

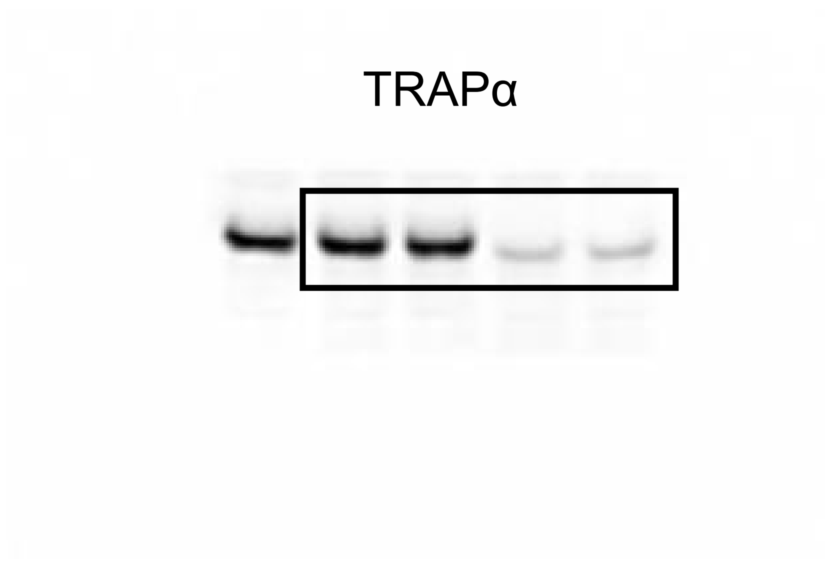


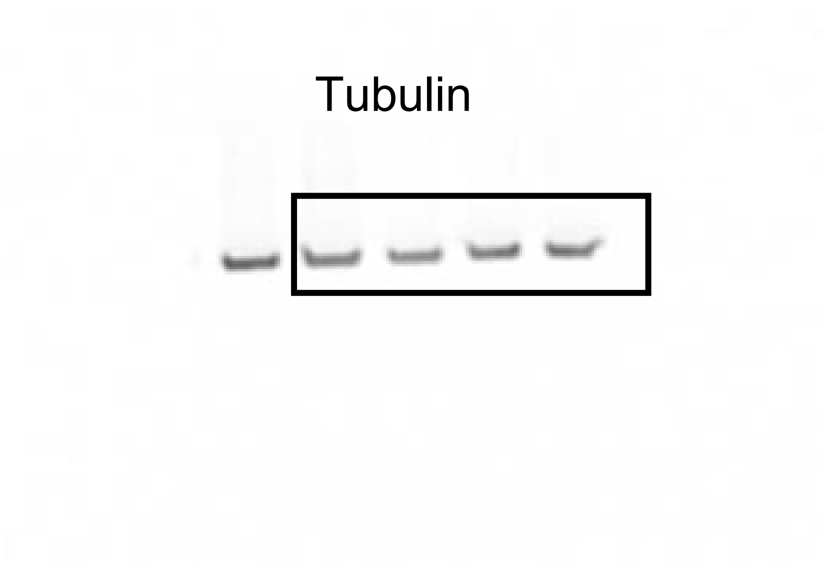

Full unedited gel for Supplemental Figure 6

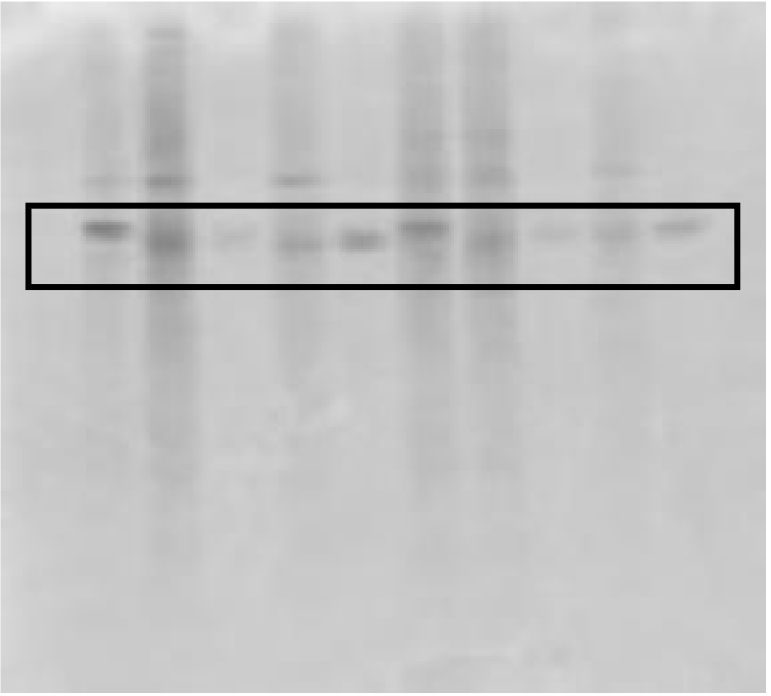

# Full unedited gel for Supplemental Figure 9

TRAPα

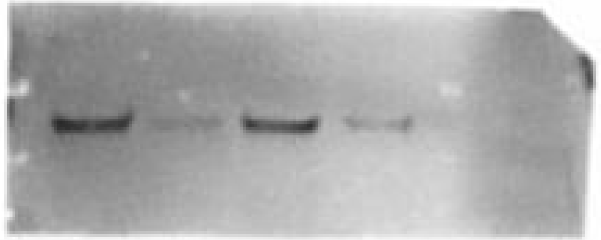

ALDH1A3

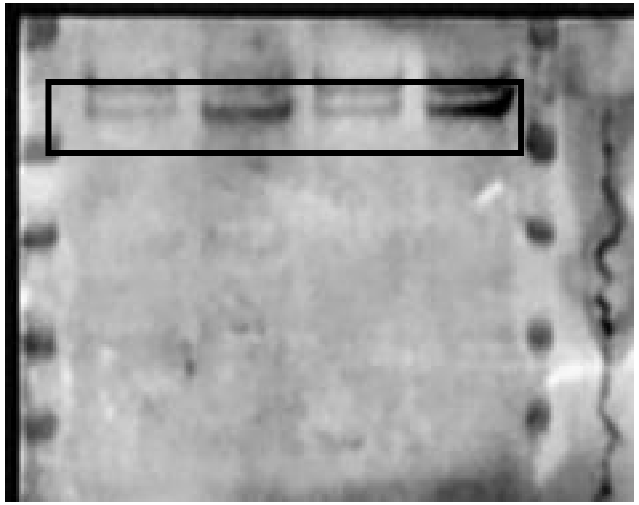

Tubulin

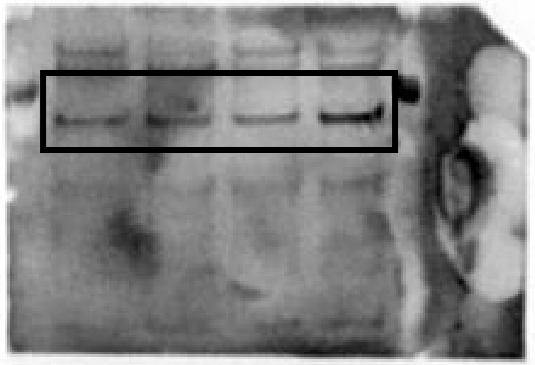

Supplement: Unedited blot and gel images [file jci-135-179845-s010.pdf]
